# A Link Between Mitochondrial Dysfunction and the Immune Microenvironment of Salivary Glands in Primary Sjogren’s Syndrome

**DOI:** 10.3389/fimmu.2022.845209

**Published:** 2022-03-14

**Authors:** Ning Li, Yusi Li, Jiawei Hu, Yicheng Wu, Jie Yang, Hongmei Fan, Lei Li, Danyang Luo, Yulin Ye, Yiming Gao, Haimin Xu, Wangxi Hai, Liting Jiang

**Affiliations:** ^1^ Department of Stomatology, Ruijin Hospital, Shanghai Jiao Tong University School of Medicine, Shanghai, China; ^2^ College of Stomatology, Shanghai Jiao Tong University, Shanghai, China; ^3^ Core Facility of Basic Medical Sciences, Shanghai Jiao Tong University School of Medicine, Shanghai, China; ^4^ Department of Pathology, Ruijin Hospital, Shanghai Jiao Tong University School of Medicine, Shanghai, China; ^5^ Department of Nuclear Medicine, Ruijin Hospital, Shanghai Jiao Tong University School of Medicine, Shanghai, China

**Keywords:** primary Sjogren’s syndrome (pSS), mitochondrial dysfunction, mitochondrial metabolism, oxidation respiratory chain, salivary gland, mitochondria, autoimmunity

## Abstract

**Background:**

Primary Sjogren’s syndrome (pSS) is a slowly progressive, inflammatory autoimmune disease characterized by lymphocytic infiltration into salivary and lacrimal glands. It becomes more recognized that morphology alterations of epithelial mitochondria are involved in altered cellular bioenergetics in pSS patients. The integrated analysis of the mitochondrial role in the pathogenesis and aberrant immune microenvironment in pSS remains unknown.

**Methods:**

The mitochondria-related genes and gene expression data were downloaded from the MitoMiner, MitoCarta, and NCBI GEO databases. We performed novel transcriptomic analysis and constructed a network between the mitochondrial function and immune microenvironment in pSS-salivary glands by computer-aided algorithms. Subsequently, real-time PCR was performed in clinical samples in order to validate the bioinformatics results. Histological staining and transmission electron microscopy (TEM) were further studied on labial salivary gland samples of non-pSS and pSS patients characterized for mitochondria-related phenotypic observation in the different stages of the disease.

**Results:**

The bioinformatic analysis revealed that the expression of several mitochondria-related genes was altered in pSS. Quantitative real-time PCR showed that four hub genes, *CD38*, *CMPK2*, *TBC1D9*, and *PYCR1*, were differentially expressed in the pSS clinical samples. These hub genes were associated with the degree of immune cell infiltration in salivary glands, the mitochondrial respiratory chain complexes, mitochondrial metabolic pathway in gluconeogenesis, TCA cycle, and pyruvate/ketone/lipid/amino acid metabolism in pSS. Clinical data revealed that the gene expression of fission (*Fis1*, *DRP1*, and *MFF*) and fusion (*MFN1*, *MFN2*, and *OPA1*) was downregulated in pSS samples, consistent with the results from the public validation database. As the disease progressed, cytochrome c and Bcl-2 proteins were regionally distributed in salivary glands from pSS patients. TEM revealed cytoplasmic lipid droplets and progressively swollen mitochondria in salivary epithelial cells.

**Conclusion:**

Our study revealed cross talk between mitochondrial dysfunction and the immune microenvironment in salivary glands of pSS patients, which may provide important insights into SS clinical management based on modulation of mitochondrial function.

## Introduction

Primary Sjogren’s syndrome (pSS) is a systemic autoimmune disease, typically presenting as keratoconjunctivitis sicca and xerostomia (1). Moreover, a significant percentage of patients are accompanied by fatigue, musculoskeletal pain, and systemic features, and complicated by lymphoma in around 2%–5% of patients (2, 3). These symptoms have a major effect on quality of life in patients with pSS (4). Accumulating evidence indicates that immune dysfunction is the main basis of the occurrence and development of pSS (5, 6). The histological hallmark of pSS is inflammatory mediators and lymphocytic infiltration to exocrine glands (1, 7). In mild lesions, the focal infiltrates around the ductal epithelium are mainly composed of CD4^+^ and CD8^+^ T lymphocytes (8). In moderate or severe lesions, a more predominant B cell environment is formed with autoantibody production. Ectopic and germinal centers presented in salivary glands caused by B cell overactivation can increase the risk for lymphoma (9).

In recent years, studies on inflammation and autoimmune disease have attracted tremendous attention (10). Under normal situations, inflammation is transient and reversible, and it protects against invasion of pathogenic microorganisms and promotes tissue repair. When a chronic inflammatory response becomes prolonged, it might lead to several chronic or autoimmune diseases (11). Clinically, pSS is characterized by overexpression of oxidative stress-related biomarkers and proinflammatory cytokines, such as tumor necrosis factor-alpha (TNF-α), interleukin-6 (IL-6), IL-12, IL-18, and gamma-interferon (12). In particular, the glandular inflammatory activity in pSS appears to be increasing linearly higher for IL-6 and IL-17 levels (10, 13). Although the exact mechanisms underlying the creation of such an inflammatory environment remain poorly understood, chronic inflammatory conditions might be activated by the disruption of cellular homeostasis, in addition to infection and injury (14).

Mitochondria are essential for maintaining cellular homeostasis, and they are metabolically active cell organelles with fine-tuned dynamics responsible for maintaining mitochondrial integrity and functions (15, 16). In addition to producing ATP, mitochondria are known as the major source for reactive oxygen species (ROS) generation through oxidative phosphorylation (OXPHOS). Damaged mitochondria generate more ROS than healthy mitochondria, which could explain abnormally elevated levels of oxidative stress markers (such as 8-OHdG) in the saliva of patients with pSS (17). Therefore, autoimmune-based mitochondrial damage related to the onset of a pro-oxidant state (overproduction of ROS) might be postulated in pSS pathogenesis (12, 15). Recent scientific advances reveal that alterations in key organelles such as mitochondria are important inflammatory triggers (18). A review by Barrera et al. suggested that release of molecular danger signals from damaged ROS-generating mitochondria triggered a potent inflammatory response *via* pattern recognition receptors (PRRs). Further, alterations with mitochondria-endoplasmic reticulum contact sites could increase inflammatory signaling (14). In addition, our previous study first reported alterations in the ultrastructure of cellular organelles in both acini and ducts from minor salivary glands, including swelling of mitochondria, and associated with disease severity (19). This mechanism is thought to be linked to chronic inflammatory and mitochondrial dysfunction in pSS. The inflammatory signaling, in turn, further amplifies the inflammatory response by recruiting immune cells. However, there are few studies on the dynamic cross talk between mitochondria in salivary gland cells and the immune microenvironment of patients with pSS.

In the current study, we combined the data of patients with pSS from the NCBI’s Gene Expression Omnibus (GEO) databases with that of the MitoMiner and MitoCarta3.0 databases to screen out differentially expressed genes (DEGs) related to mitochondria. Notably, a novel transcriptomic analysis was performed to construct a network between mitochondrial function and immune microenvironment in pSS-salivary glands by computer-aided algorithms. Furthermore, we observed the mitochondria-related genetic and phenotypic changes on labial salivary glands (LSGs) from patients with non-pSS and pSS patients to visualize the severity of disease. The exploration of changes in mitochondrial function in salivary glands of patients with pSS introduces new insights into potential therapeutic targets and clinical management.

## Materials and Methods

### Patients and Labial Minor Salivary Gland Biopsy

LSG biopsies from 48 patients with pSS (without any related treatment) and 12 age- and gender-matched non-pSS sicca controls were performed in this study. The diagnosis of pSS was fulfilled according to the 2016 American College of Rheumatology/European League Against Rheumatism classification criteria (20) or the 2012 ACR classification criteria (21). Patients with non-pSS met the same diagnostic criteria as patients presenting with xerostomia and xerophthalmia but did not meet the classification criteria for pSS. Clinical information and samples were collected after patients signed written informed consent. The complete details are shown in **Supplementary Table S1**. The Ethics Committee of Ruijin Hospital, Shanghai Jiao Tong University School of Medicine and Chinese Clinical Trial Registry, approved the study (ChiCTR2000039820). The patients with pSS were further stratified into two distinct stage groups, according to the severity of lymphocyte infiltration foci in labial salivary gland biopsy (13, 22). Patients with mild lesions (focal lymphocytic sialadenitis (FLS), with focus scores (FS) <2) were included in the low-infiltration stage (pSS1), and patients with severe lesions (FLS, with FS ≥2) were included in the high-infiltration stage (pSS2).

### Histological Staining

For histological staining, LSG samples were fixed freshly in 4% neutral formaldehyde overnight and embedded in paraffin. Samples were cut into 5-μm-thick serial sections. Immunohistochemical staining (IHC) was performed according to the manufacturer’s instructions. Briefly, slides were deparaffinized and microwave heated in citrate buffer (pH 6.0) to retrieve antigen. After gradual chilling, endogenous peroxidase activity was quenched using 3% hydrogen peroxide. Protein blockage was applied using 3% BSA for 30 min before incubation with primary antibodies at 4°C overnight. After washing with PBS, slides were incubated with secondary antibodies for 50 min at room temperature. Slides underwent color development with DAB (K5007, Dako, Denmark) followed by counterstaining in hematoxylin. The following primary antibodies were used: cytochrome c (1:500, ab133504, Abcam, UK) and Bcl-2 (IR614, Dako, Denmark). Finally, the slides were visualized under a light microscope (Nikon Eclipse Ni-U, Japan), and the images were captured using a camera attached to the microscope.

### Transmission Electron Microscopy

The ultrastructure of the minor salivary glands from non-pSS and pSS patients was visualized by transmission electron microscopy (TEM). Following fixation with 2.5% glutaraldehyde, the samples were postfixed in 1% osmium tetroxide and dehydrated using a gradient series of ethyl alcohol. Samples were then embedded in Embed 812 resin (EMS, TED PELLA, USA) and propylene oxide solutions followed by embedding in embedding resin for 48 h. The blocks were sectioned transversely at 70–90 nm using a diamond knife (EM UC7; Leica, Wetzlar, Germany). Ultrathin sections were stained with lead citrate and photographed with a transmission electron microscope (H-7650; Hitachi, Tokyo, Japan).

### Data Acquirement and Preprocessing

**Figure 1:**
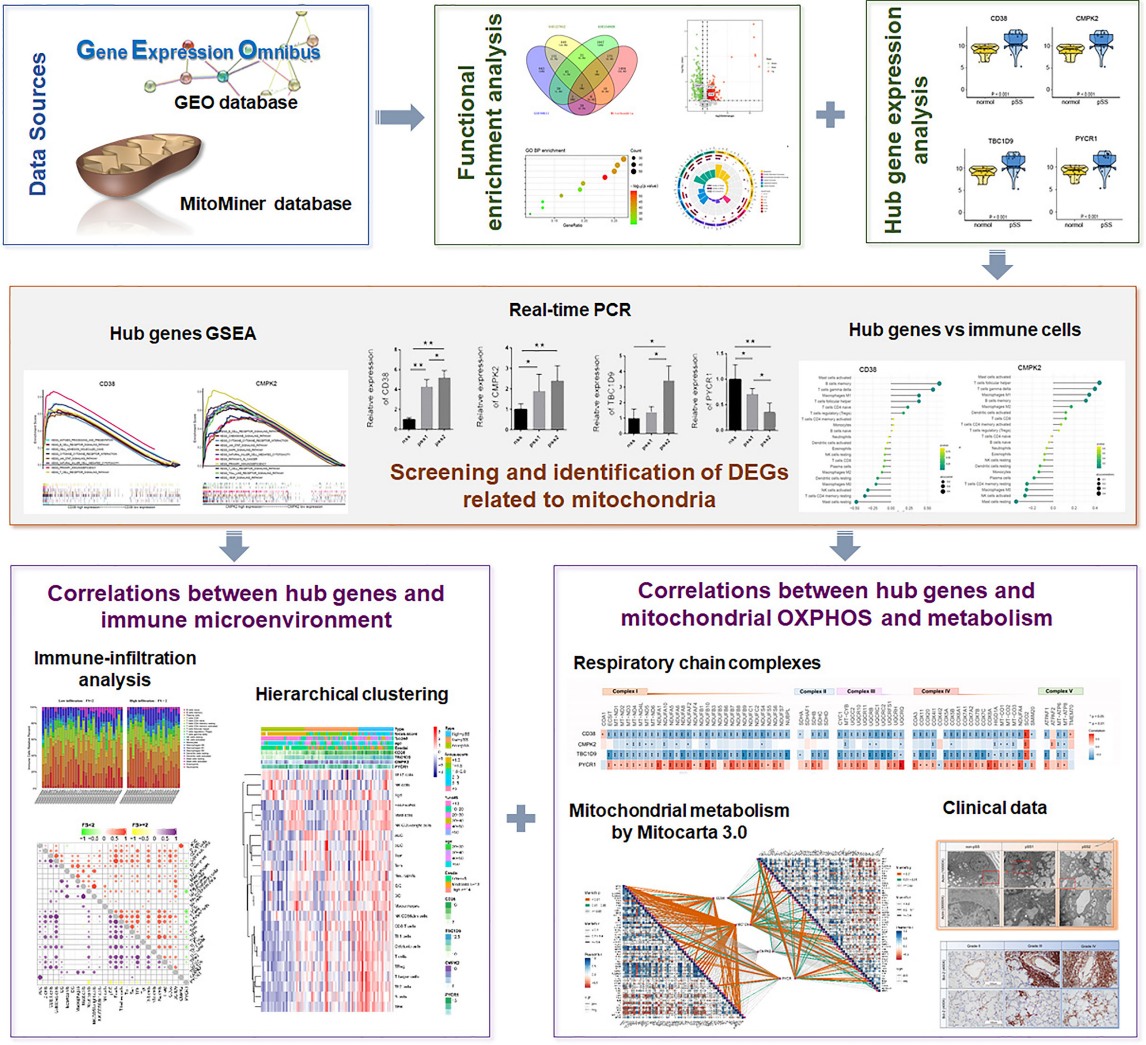
The illustrations for this study. The overall protocol utilized in the current study to construct a network between mitochondria and immune microenvironment in primary Sjogren’s syndrome (pSS).

The integration of our clinical data and bioinformatic analyses is illustrated by the flowchart in **Figure 1**. The pSS cohorts with publicly available datasets were obtained from GEO databases (23), including GSE40611 (24), GSE127952 (https://www.ncbi.nlm.nih.gov/geo/query/acc.cgi?acc=GSE127952), and GSE154926 (https://www.ncbi.nlm.nih.gov/geo/query/acc.cgi?acc=GSE154926). For validation, RNA-seq data and clinical information of an additional 39 patients with pSS were obtained from another publicly available dataset GSE173808 (25). We stratified the patients into three distinct groups: non-pSS (n = 12 labial glands, n = 14 parotid glands), pSS-low infiltration (focus score (FS) <2, n = 18 labial glands, n = 14 parotid glands), and pSS-high infiltration (FS ≥2, n = 14 labial glands, n = 7 parotid glands) (13). The Limma package and “DESeq2” package of R v3.6.1 were used for array data sets and high-throughput sequencing count data standardization, respectively, and the standardized matrix file is obtained. When multiple transcript IDs were present for one gene, we chose the ID with the highest average expression. Raw gene expression data were log2 transformed and quantile-normalized for all subsequent downstream analyses.

### Identification of Mitochondria-Related DEGs and Functional Enrichment Analysis

DEGs were selected using the “limma” and “DEseq2” R packages with a maximum posteriori absolute log2|fold-change| ≥1 and a Benjamini–Hochberg *p*-value <0.05. The Integrated Mitochondrial Protein Index (IMPI) of the MitoMiner database (http://mitominer.mrc-mbu.cam.ac.uk/) provides 1626 human genes that encode mitochondrially localized proteins (26). Overlapping mDEGs based on the MitoMiner database were extracted from GSE40611, GSE127952, and GSE154926, respectively, and visualized with a heatmap, Venn diagram, and volcano plot. The list of DEGs was used for GO (http://geneontology.org/docs/go-citation-policy/) and KEGG enrichment analyses (https://www.genome.jp/kegg/kegg1.html) (27), using the clusterprofiler package of R software (28, 29). Bioinformatic pathway analysis was conducted with the Gene Set Enrichment Analysis (GSEA) (https://www.broadinstitute.org/gsea/). GSEA is a computational method to detect statistical significance, and pathways using the KEGG gene set (c2.cp.kegg.v7.4.symbols.gmt) from the Molecular Signatures Database (MSigDB) (http://software.broadinstitute.org/gsea/msigdb/) by the JAVA program were selected as the reference gene sets (30). The algorithm of random sampling was 1,000 permutations. Only enrichment pathways with p < 0.05 and false discovery rate < 0.05 were considered statistically significant.

### Hub Genes and pSS-Infiltrating Immune Cell Analysis

Heatmaps of the mDEGs were generated by “pheatmap” package v1.0.8 (https://CRAN.R-project.org/package=pheatmap) of R. To further investigate the correlation between hub genes and immune cell infiltration, formatted data were uploaded to the Cell-type Identification By Estimating Relative Subsets Of RNA Transcripts (CIBERSORT) R program (http://cibersort.stanford.edu). The CIBERSORT algorithm is a deconvolution algorithm that has been validated on gene expression profiles measured by RNA-sequencing, and it derives a *p*-value for the deconvolution of each sample with *p* < 0·05 considered accurate (31). We used CIBERSORT to estimate the fraction of 22 immune cell types in salivary glands from patients with pSS (GSE154926). The correlation between each hub gene and the 22 immune cells was tested by Spearman’s rank correlation and presented as a lollipop chart. We also performed functional enrichment analyses using the KEGG pathway dataset from GSEA.

Marker genes for immune cell types were identified (32). Of 24 gene signatures, 11 [e.g., dendritic cells (DCs), eosinophils, mast cells, macrophages, neutrophils, and natural killer cells (NKs)] were for immune cells in adaptive immunity, 13 (e.g., B, CD8^+^ T, T helper 1 [T_h1_], T_h2_, central memory T [T_cm_], effector memory T [T_em_], regulatory T [T_reg_], and T follicular helper [T_fh_] cells) for innate immunity. The immune infiltration score was calculated using the single-sample GSEA (ssGSEA) method (33) based on the GSE173808 dataset. Changes in the immune score between high and low hub gene expression subgroups were analyzed using the “ggpubr” package (https://github.com/kassambara/ggpubr) *via* a Wilcox test. Heatmaps and clustering analyses were generated using the “ComplexHeatmap” v2.10.0 package in R to show the correlation (34). Spearman correlation based on the genes and immune cells of interest was performed using the “pheatmap” package.

### Bioinformatic Evaluation of Mitochondrial Respiratory Chain and Mitochondrial Metabolism in pSS

From the MitoCarta3.0 database (35) and high-throughput sequencing data (GSE173808), the DEGs of the oxidative respiratory chain complex were computed using the “limma” package and visualized with the “pheatmap” R package. The correlation between the four hub genes and five oxidative respiratory chain complex genes was calculated using Spearman’s rank correlation and visualized using the “ggplot2” R package (https://cran.r-project.org/web/packages/ggplot2/ggplot2.pdf) (36). We assessed the interrelationship between four hub genes and selected OXPHOS genes using Pearson’s correlation (R) with the function scatter from the “ggpubr” R package v0.4.0 (https://CRAN.R-project.org/package=ggpubr).

Correlations between four hub genes and mitochondrial metabolism, damage-associated molecular patterns (DAMPs) were computed with the Mantel test (37) and the Pearson correlation coefficient in pSS-low-infiltration and pSS-high-infiltration groups. The “ggcor” R package v0.9.8.1 (https://github.com/houyunhuang/ggcor), based on “ggplot2,” was used to provide a graphical display of any correlations and their combinations. In addition, we analyzed the expression of DAMP-related genes (*NLRP3*, *ZBP1*, *TNF*) and apoptosis-related genes (*Bcl-2*, *Bax*, *caspase3*) using the “ggpubr” package *via* a Wilcox test.

### Quantitative Real-Time PCR

The LSG samples collected from patients were immediately immersed in the Allprotect™ Nucleic Acid and Protein Stabilization Reagent (R0121, Beyotime, Shanghai, China). Then, RNA was extracted and first-strand cDNA synthesis was performed using PrimeScript™ RT Master Mix (No. RR036A, Takara, Shiga, Japan), and qPCR was performed by TB Green^®^ Premix Ex Taq™ II (No. RR420A, Takara, Shiga, Japan). Primer sequences are summarized in **Supplemental Table S2**, and *β-actin* was applied as an internal reference. The relative expression of target genes was calculated by the 2^−ΔΔCt^ method. All PCR reactions were conducted in triplicate.

### Statistical Analysis

Quantitative result data are presented as means ± SD. Statistical analysis was carried out with Student’s *t*-test or one-way analysis of variance in GraphPad Prism software. Statistical significance was set at *p <*0.05.

## Results

### Identification of Mitochondria-Related DEGs and Functional Enrichment Analysis in pSS

**Figure 2:**
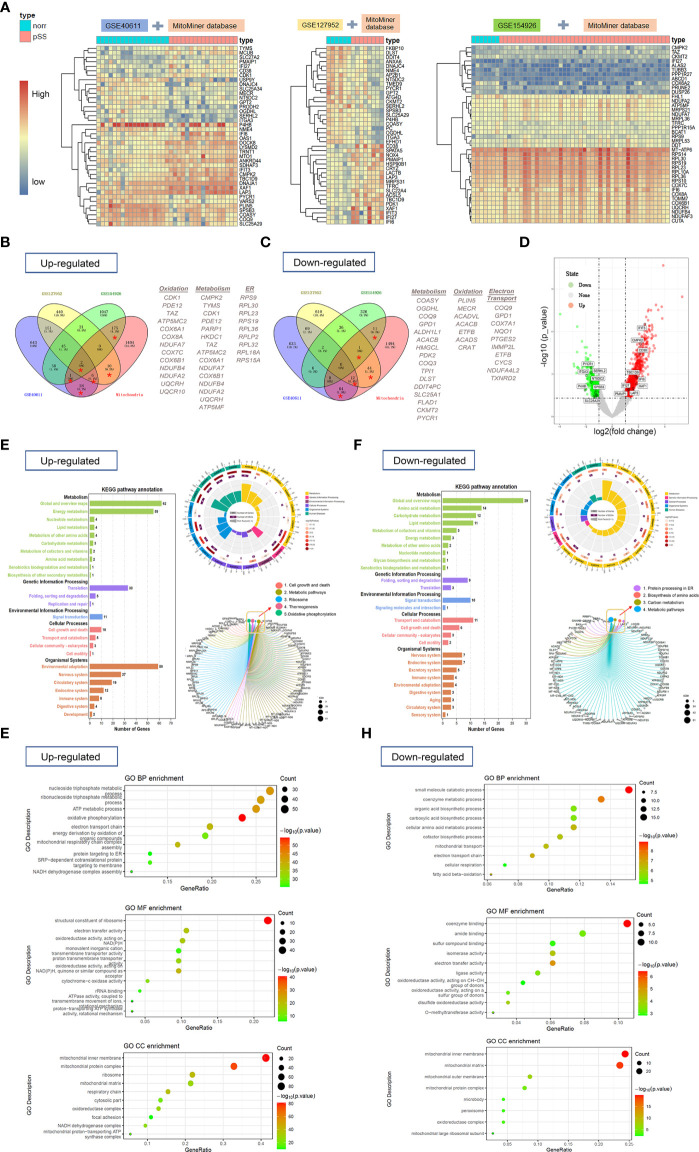
The heat maps, Venn diagram, and volcano plot of mitochondria-related-DEGs and gene-set enrichment analysis (NCBI-GEO database). **(A)** The heat maps showed overlapping DEGs based on the MitoMiner database which were extracted from 3 publicly available GEO datasets by unsupervised hierarchical clustering within three groups. R-package heatmap was used for figure generation. **(B, C)** Venn diagrams showed the number of upregulated **(B)** and downregulated DEGs **(C)** for each dataset and the number of genes that overlap between them. **(D)** Volcano plot showed mitochondria-related DEGs (≥2 times of intersection) between pSS and controls. **(E, F)** The KEGG pathway analyses of upregulated **(E)** and downregulated DEGs **(F)** were performed with R package clusterProfiler and GSEA based on MSigDB C2-curated KEGG gene sets. **(G, H)** GO analysis of upregulated **(G)** and downregulated **(H)** mitochondria-related DEGs was performed to identify enriched biological process including molecular function, cell component, and categories.

In this study, three publicly available datasets—GSE40611, GSE127952, and GSE154926—which contained 17, 8, and 43 patients with pSS were used as training datasets. In addition, the GSE173808 dataset which includes 39 patients with pSS was applied as a validation cohort. We then analyzed the DEGs associated with mitochondria based on the MitoMiner database. Heatmaps representing the most significant mDEGs (log2|fold-change| >1) are shown in **Figure 2A** and revealed a clear distinction between patients with or without pSS. **Figures 2B, C** show Venn diagrams representing the overlap between these mDEGs. A volcano plot represents the mDEGs (≥2 times intersection) between patients with pSS and controls. The top upregulated DEGs included IFIT3, CMPK2, and CD38 (**Figure 2D**).

Functional analysis of upregulated mDEGs revealed enrichment in KEGG pathways related to metabolism, translation, cell growth and death, ribosome, thermogenesis, and OXPHOS in patients with pSS (**Figure 2E**). The downregulated KEGG pathways involved protein processing in the endoplasmic reticulum, biosynthesis of amino acids, carbon metabolism, and metabolic pathways (**Figure 2F**). In addition, three domains of gene ontology (GO; biological process, molecular function, and cellular component) were analyzed using the GO database. The mDEGs upregulated for pSS were related to the biological processes OXPHOS, nucleoside triphosphate metabolic process, and adenosine triphosphate (ATP) metabolic process. Among the most relevant downregulated BP terms were that of the small-molecule catabolic process, co-enzyme metabolic process, electron transport chain, and fatty acid beta-oxidation (**Figures 2G, H**). The complete details are shown in **Supplementary Table S3**.

### Identification and Validation of Mitochondria and Immune-Related Hub Genes

**Figure 3:**
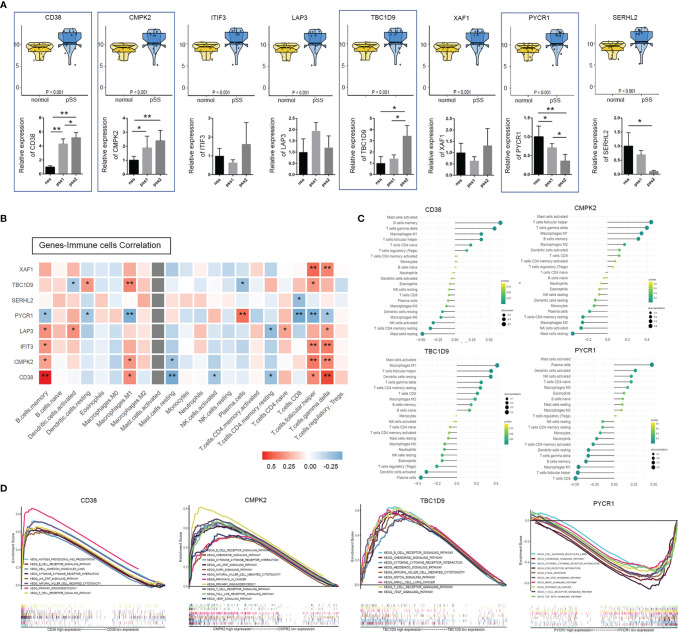
Identification and validation of mitochondria & immune-related hub genes. **(A)** Top: expression analyses of the 8 mitochondria-related genes (CD38, CMPK2, ITIF3, LAP3, TBC1D9, XAF1, PYCR1, and SERHL2) in normal and pSS patient cohorts were illustrated using the R package “ggpubr” function (p < 0.001, Wilcox test) (NCBI-GEO database). Bottom: above genes identified by quantitative real-time PCR from our own cohort. Data shown were normalized to *Actin* expression and were relative to expression in the non-pSS (n = 3, error bars represent mean ± SD, *p < 0.05, and **p < 0.01 by Student’s *t* test). **(B)** Heatmap shows Spearman correlation between hub genes and immune cells. **(C)** The lollipop chart of CD38, CMPK2, TBC1D9, and PYCR1 demonstrates the correlation between genes and immune cells, an extension of **Figure 4B**. Lollipop size corresponds to the strength of this correlation. **(D)** Gene Set Enrichment Analysis (GSEA) of KEGG pathway enrichment for CD38, CMPK2, TBC1D9, and PYCR1 high-expression group versus low-expression group.

The patients from the GSE154926 dataset were further used to screen out a mitochondrial-related gene signature. The expression heatmaps were generated by the “pheatmap” R package, and 21 mitochondrial-related genes (*CD38*, *CMPK2*, *ITIF3*, *LAP3*, *TBC1D9*, *XAF1*, *IFI6*, *PMAIP1*, *IFI27*, *COASY*, *DNAJC4*, *GPT2*, *ITGA3*, *PYCR1*, *SERHL2*, *NME4*, *NT5DC2*, *SLC25A29*, *OGDHL*, *P4HB*, *SPSB3*) were identified (*p* < 0.05). Among them, eight genes (*p* < 0.001) were further verified by real-time PCR (**Figure 3A**). The results suggested that the genes *CD38*, *CMPK2*, and *TBC1D9* were relatively overexpressed in LSGs from all patients with pSS, while *PYCR1* was underexpressed (*p* < 0.05). To further investigate the relationship between hub genes and infiltration of immune cells, the CIBERSORT algorithm was used on data of patients with pSS. As shown in **Figures 3B, C**, *PYCR1* had a significant negative correlation with infiltration of memory B cells, M1 macrophages, CD8^+^ T cells, and T_fh_ (*p* < 0.05), while *CD38* and *CMPK2* had an opposite trend (*p* < 0.05). In addition, *TBC1D9* had a positive correlation with infiltration of resting DCs, M1 macrophages, and T_fh_ but a negative correlation with plasma cells (*p <*0.05). In contrast, *PYCR1* showed an opposite trend (*p* < 0.05). Subsequently, a total of four mitochondria-related genes (*CD38*, *CMPK2*, *TBC1D9*, *PYCR1*) were identified as the most promising factors associated with pSS disease severity.

GSEA was used to obtain a deeper insight into the function of DEGs. As shown in **Figure 3D**, overexpression of hub DEGs (*CD38*, *CDMK2*, *TBC19*) was highly enriched in pathways related to T cell/B cell receptor signaling pathways, primary immunodeficiency, NK cell-mediated cytotoxicity, cytokine–cytokine receptor interaction, and the JAK/STAT signaling pathway (*p* < 0.05). The pathways altered by *PYCR1* were involved in the T cell receptor signaling pathway (*p* < 0.05, FDR 0.079), cytokine–cytokine receptor interaction (*p* < 0.01, FDR 0.049), and the JAK/STAT signaling pathway (*p* < 0.01, FDR 0.051) (**Supplemental Table S4**). Interestingly, the GSEA results confirmed a strong association between the above four mitochondria-related genes and immune-related signaling pathways.

### Immune Cell Infiltration and Association Between Hub Genes and Immune Microenvironment

**Figure 4:**
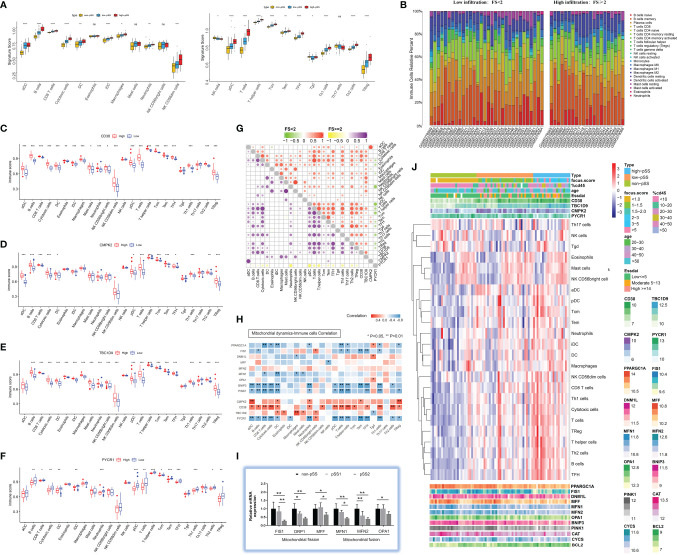
Characterization of immune infiltration in salivary glands in pSS. **(A)** Boxplot of the normalized signature score based on single-sample gene set enrichment analysis (ssGSEA) in the validation cohort. The yellow boxplot represents non-pSS patients, the blue boxplot represents low-infiltration pSS patients, and the red boxplot represents high-infiltration pSS patients. ***p < 0.001, **p < 0.01, *p < 0.05, ns = 1 (independent sample Kruskal–Wallis test). **(B)** Comparisons of immune-cell proportion between pSS-low-infiltration and pSS-high-infiltration groups in the validation cohort. (C–F) Comparison of 24 immune cell infiltration for CD38 **(C)**, CMPK2 **(D)**, TBC1D9 **(E)**, and PYCR1 **(F)** high-expression groups versus low-expression group. **(G)** Spearman correlation between 4 hub genes (CD38, CMPK2, TBC1D9, and PYCR1) and 24 immune cells in pSS-low-infiltration and pSS-high-infiltration groups. **(H)** Correlation between interested genes (4 hub genes and mitochondrial dynamic-related genes) and 24 immune cells in pSS patients. **(I)** The expression profile of genes regulating mitochondrial dynamics was evaluated by quantitative real-time PCR from our own cohort. Data shown are normalized to *Actin* expression and are relative to expression in the non-pSS (n = 3, error bars represent mean ± SD). *p < 0.05, and **p < 0.01 by Student’s *t* test. **(J)** Heatmap shows the enrichment scores of interested genes (4 hub genes and mitochondrial dynamic-related genes) and immune cells in pSS samples. Each column represents an individual patient sample, and each row represents a gene or an immune cell, ordered by unsupervised hierarchical clustering.

The relative level of immune cell infiltration for each patient (data from publicly available datasets mentioned above) was investigated for pSS. We compared the signature score of 24 immune cells using ssGSEA in patients stratified by histological focus score (non-pSS (FS = 0) vs. pSS-low infiltration (FS < 2) vs. pSS-high infiltration (FS ≥ 2)) depending on the validation cohort. The Kruskal–Wallis test revealed that the DC family (activated DC, immature DC, DC, plasmacytoid DC [pDC]), B cells, CD8^+^ T cells, cytotoxic cells, NK cells, T_h1/2_ cells, T_fh_, and T_reg_ cells were significantly higher in the pSS-high-infiltration group (*p* < 0.05) (**Figure 4A**). The bar plots in **Figure 4B** show the proportion of 22 immune cells in the pSS1 and pSS2 groups. The six most common immune cells in the pSS-high filtration group were memory B cells (26.7%), resting memory T cells CD4^+^ (15.2%), plasma cells (13.7%), M2 macrophages (10.3%), CD8^+^ T cells (9.2%), and M0 macrophages (7.8%), while the six most common immune cells in the pSS-low-filtration group were plasma cells (23.6%), resting memory B cells (17.4%), resting memory CD4^+^ T cells (16.0%), M2 macrophages (9.6%), naïve B cells (7.6%), and CD8^+^ T cells (7.0%). As shown in **Figures 4C–F**, there was some evidence of higher B cells, CD8^+^ cells, cytotoxic cells, T cells, T_cm_, T_em_, and T_reg_ immune scores for high *CD38*, *CMPK2*, and *TBC1D9* expression (*p* < 0.001), while *PYCR1* showed an opposite trend (*p* < 0.05, Wilcox test). In the high-immune cell infiltration group, *CD38* was also positively correlated with B cells, CD8^+^ T cells, and cytotoxic cells, while *PYCR1* was negatively correlated with cytotoxic cells, pDC, neutrophils, and T cells (**Figure 4G**).

### Mitochondrial Dysfunction in pSS Including Abnormal Mitochondrial Dynamics, Impaired Mitochondrial Respiratory Chain Function, and Damaged Mitochondrial Metabolism

#### Mitochondrial Dynamics and Immune Cells

Mitochondrial dynamics is regulated by fusion and fission proteins (38). Mitochondrial fusion is usually protective, and mitochondrial fission is crucial to clearing the damaged mitochondria by mitophagy. Repeated cycles of fusion and fission facilitate the sharing of mitochondrial genetic content, ions, metabolites, and proteins (14). Therefore, to probe the relationship between mitochondrial dynamics and the immune microenvironment in pSS was warranted. As is shown in **Figure 4H**, the mitochondrial biogenesis (*PCG-1α*) and mitophagy (*BNIP3/PINK1*) markers were negatively correlated with CD8^+^ T cells, cytotoxic cells, DCs, neutrophils, T cells, T helper cells, and T_h1_ cells (*p* < 0.05). *Fis1* was negatively related to B, T, T helper, T_em_, T_fh_, and T_h1_ cells, while *MFN1* was negatively related to CD8^+^ T cells, DCs, macrophages, and mast cells (*p* < 0.05). For a more quantitative analysis of mitochondrial dynamics in LSGs, we performed real-time PCR on whole gland lysates using specific primers for mitochondrial fission (*FIS1*, *DRP1*, *MFF1*) and fusion (*MFN1*, *MFN2*, *OPA1*) markers. The results indicated a significant decrease in mitochondrial fission and fusion markers in pSS2 compared with non-pSS (*p* < 0.05) (**Figure 4I**), which is consistent with the results of the validation dataset (**Figure 4J**).

#### Mitochondrial Respiratory Chain and Immune Cells

**Figure 5:**
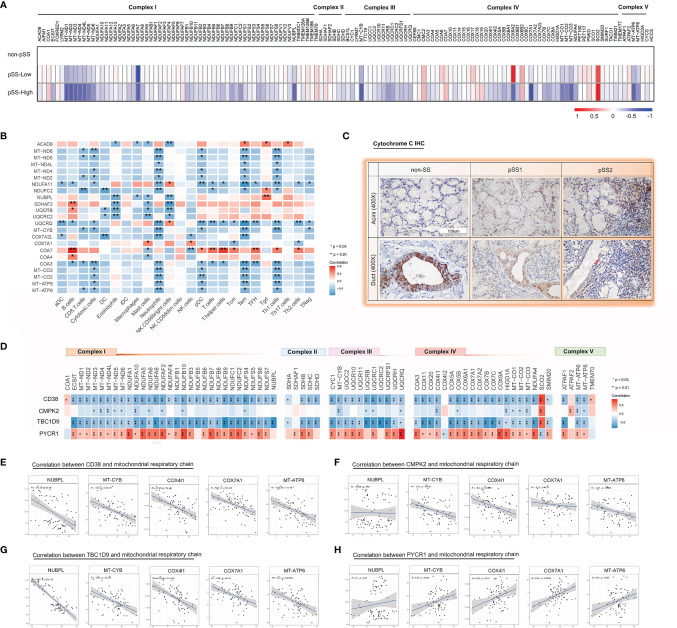
Impaired mitochondrial respiratory chain complexes in the salivary glands from pSS. **(A)** A heatmap shows significant down regulation in the bulk respiratory chain complex (I–V) genes in pSS from the validation cohort, particularly those encoded in the mitochondria in pSS-high-infiltration group. **(B)** Heatmap of the Spearman correlation values between respiratory chain complex (I–V) genes and immune responses in pSS from the validation cohort. **(C)** Immunohistochemical staining for cytochrome c of paraffin-embedded labial salivary gland specimens from our own cohort. Scale bar = 100 μm. **(D)** Heatmap of the Spearman correlation values between respiratory chain complex (I–V) genes and 4 hub genes (CD38, CMPK2, TBC1D9, and PYCR1) in pSS from the validation cohort. (E–H) Scatter plot depicted the correlation between interested respiratory chain complex genes (MT-CYB, MT-ATP6, COX7A1, NUBPL, COX4I1, CYCS) and CD38 **(E)**, CMPK2 **(F)**, TBC1D9 **(G)**, and PYCR1 **(H)** based on the data from **Figure 6D**, respectively.

The main role of mitochondria is to convert nutrients to ATP *via* the process of OXPHOS, which is regulated through four respiratory chain complexes (I–IV) and ATP synthase (complex V) (39). In this study, a publicly available pSS cohort was included for a better understanding of pSS-related effects on the expression of respiratory chain-related genes. Heatmaps using the “pheatmap” R package revealed downregulation of multiple nuclear and mitochondrial DNA encoded genes, particularly those in the pSS-high-infiltration group from the GSE173808 dataset (**Figure 5A**). As shown in **Figure 5B**, the mitochondrial genes (*MT-ND2*, *MT-ND5*, *MT-ND6*, *MT-CYB*) and nuclear genes (*COA3*), which belong to proton-pumping complexes (I, III, IV), had a negative correlation with CD8^+^ T cells, cytotoxic cells, neutrophils, pDC, T_em_ cells, and T_h1_ cells. Two complex I genes (*ACAD9*, *NUBPL*) and three complex II genes (*SDHAF2*, *UQCRB*, *UQCRC2*) had a negative correlation with eosinophils, mast cells, and CD56^bright^ NK cells. Spearman correlation values revealed that there was a clear negative correlation of respiratory chain complex (I–V) genes with *CD38* and *TBC1D9* expression levels and a positive correlation with the *PYCR1* expression level in pSS (**Figure 5D**). The “ggpubr” R package was used to further assess the correlation values between four hub genes (*CD38*, *CMPK2*, *TBC1D9*, *PYCR1*) and the respiratory chain complex genes (*MT-CYB*, *MT-ATP6*, *COX7A1*, *NUBPL*, *COX4I1*, *CYCS*) of interest, as shown in **Figures 5E–H**. Collectively, these results suggested that the mitochondrial respiratory chain was gradually damaged associated with a higher degree of immune-cell infiltration.

**Figure 6:**
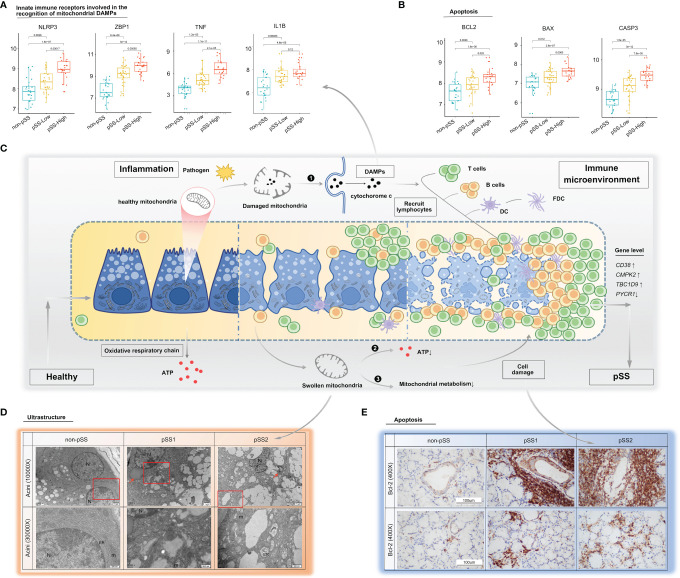
Mitochondria as a possible link between inflammation and immune microenvironment in pSS. **(A, B)** Differential gene expression analysis of damage-associated molecular patterns **(A)** and apoptosis **(B)** was conducted using the ggpubr package *via* the Wilcox test from the validation cohort. **(C)** Schematic diagram describing possible mechanisms on how mitochondria act as a bridge between inflammation and immune microenvironment in pSS. The inflammatory environment in salivary glands of pSS initiated by a multitude of promising factors, including pathogen, which can indirectly or directly cause mitochondrial dysfunction. (❶ Once cell damages, some molecules, such as cytochrome c, carried by mitochondria will be released into inappropriate compartments and serve as DAMPs, recognized by immune cells, and trigger immune responses; ❷ impaired five mitochondrial respiratory-chain complexes had reduced ATP production level through OXPHOS, which often lead to aggravated cell damage; and ❸ damaged mitochondria had been associated with abnormal mitochondrial energy metabolism, and it might further compromise epithelia cell survival. Consequently, a vicious pathological circle is activated, consisting of DAMP release, massive immune cell infiltration, and mitochondrial damage.) DAMPs: damage-associated molecular patterns, OXPHOS: oxidative phosphorylation system. **(D)** TEM of labial salivary gland tissue from our own cohort shows cytoplasmic lipid droplets (red arrows) and progressive swollen mitochondria in salivary epithelial cells as disease progress (red frame). **(E)** Immunohistochemical staining for Bcl-2 of paraffin-embedded labial salivary gland specimens. Scale bar = 100 μm.

Furthermore, in the mitochondrial respiratory chain, cytochrome c is an important component responsible for 90% of cellular oxygen consumption in mammals. Moreover, cytochrome c release is regulated by permeabilization of the mitochondrial outer membrane controlled by Bcl-2 proteins during apoptosis (40). To further investigate cytochrome c and Bcl-2 localization in the LSGs of patients, we performed IHC with specific antibodies. The results indicated that cytochrome c is primarily localized to the cytoplasm in normal salivary duct epithelial cells, with lower levels in acini tissues (**Figure 5C**). Bcl-2 was diffusely localized in the cytoplasm with lower levels in normal salivary epithelial cells (**Figure 6E**). Interestingly, we observed cytochrome c and Bcl-2 with clear regionally specialized distribution as the disease worsened. In pSS2, cytochrome c levels were low in damaged ductal cells but highly abundant in the interstitial region infiltrated by abundant lymphocytes (**Figure 5C**).

#### Mitochondrial Metabolism

**Figure 7:**
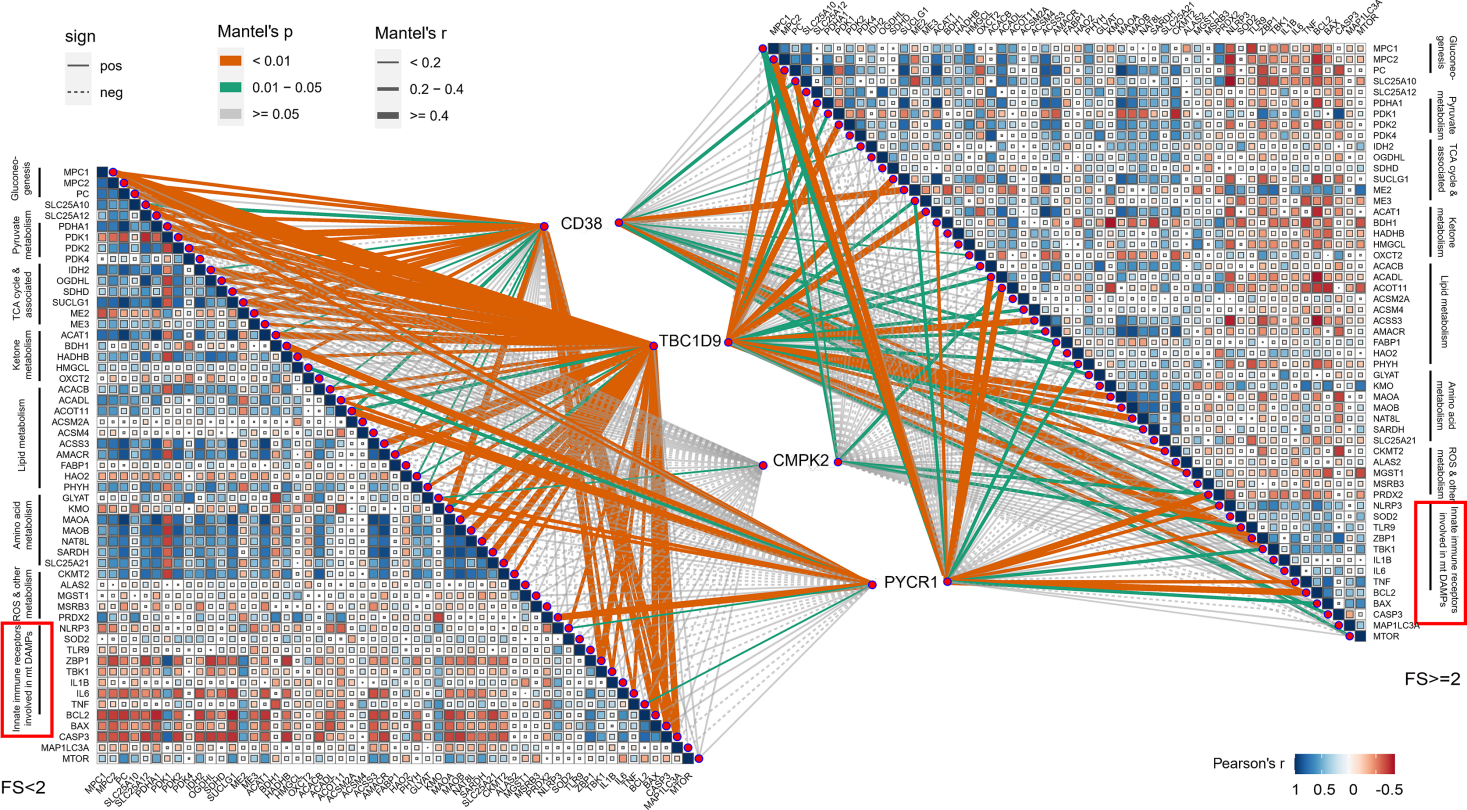
Correlations between 4 hub genes and mitochondrial metabolism, damage-associated molecular patterns (DAMPs). Color represents Pearson’s correlation coefficient r of each hub genes (CD38, CMPK2, TBC1D9, and PYCR1) versus mitochondrial metabolism-related genes and DAMP gene expression, with blue color indicating a positive correlation (Pearson’s r < 0), red color indicating a negative correlation (Pearson’s r > 0). Statistical analysis was done with the Mantel test, with yellow line indicating p value <0.01, green line indicating 0.01 < p < 0.05.

The mitochondrial impairment could also be indicated *via* reduced expression of mitochondria-related genes from the mitochondrial metabolic pathway. To further investigate the potential relevance of the four hub genes (*CD38*, *CMPK2*, *TBC1D9*, *PYCR1*), DAMPs, and mitochondrial metabolism, we used the Mantel test to analyze the statistical significance and visualize the results using the “ggcor” R package. We confirmed that they were closely related to the mitochondrial metabolic pathway in gluconeogenesis, TCA cycle, and pyruvate/ketone/lipid/amino acid metabolism in pSS (Mantel’s *p <*0.05, Pearson’s correlation, **Figure 7**). DAMPs are known as intracellular components such as endogenous proteins released from dying or dead cells during inflammation. When salivary gland tissue is damaged, DAMPs might be increasingly formed or released from epithelial cells, and the elevated extracellular DAMPs could recruit and activate immune cells (14). The transcriptional analysis revealed that the mRNA levels of *NLRP3*, *ZBP1*, *TNF*, and *IL-1β* were upregulated with increased lymphocyte infiltration (**Figure 6A**), which could be negatively related to mitochondria gluconeogenesis, ketone metabolism, and lipid metabolism in pSS (**Figure 7**). In the high-immune cell infiltration group, TEM of LSG tissues showed cytoplasmic lipid droplets and progressively swollen mitochondria in salivary epithelial cells (**Figure 6D**). A significant increase in the Bcl-2 level was observed in the region of damaged ductal cells with lymphocyte infiltration (**Figure 6E**), consistent with the bioinformatics results (**Figure 6B**).

## Discussion

Constant efforts to understand salivary gland biology have allowed us to improve our knowledge of this complex molecule. The findings in this study provide unique insights into the pathobiology of mitochondrial function and immune infiltration in pSS. We screened and identified four mitochondria-related DEGs (*CD38*, *CMPK2*, *TBC1D9*, and *PYCR1*) based on public databases and our clinical data. Especially, expression levels for *CD38*, *CMPK2*, and *TBC1D9* were elevated in the pSS group. It is remarkable that CD38 may facilitate the development of inflammatory and autoimmune diseases by regulating immune response (41), and CMPK2 is reported to control NLRP3 inflammasome activation (42). TBC1D9 appears to be a specific regulator in response to Ca^2+^ signaling and could regulate TBK1 activation associated with autoimmune disease (43). These findings may help explain the above results. In addition, our work further evaluated the mitochondrial dynamics, respiratory chain function, and metabolism response to changes in the immune microenvironment from the salivary glands of pSS, which revealed the relationship between mitochondrial dysfunction and inflammatory immune response in pSS. Therefore, protecting mitochondrial function might be effective interventions in the treatment of pSS.

The pathogenic role of the immune microenvironment in controlling pSS progression has been widely researched (9, 44). An earlier study investigated the distribution of major types of infiltrating immune cells in pSS-minor salivary gland lesions on pSS severity (9). Until now, the composition of the immune infiltrates of pSS-salivary glands has been investigated mainly with histological staining. With the recent advances in next-generation sequencing (NGS), RNA-Sequencing (RNA-seq) and computational approaches provide an unprecedented analysis of such transcripts describing immune cellular components based on publicly available immune-specific marker gene sets (32, 45). In the current study, we applied and proposed a computational approach based on CIBERSORT or the ssGSEA algorithm to deconvolute pSS-cell types from available RNA-seq data. In line with previous studies used IHC, we reported that the extent of immune cell infiltrates was well correlated with disease state. T cells, (mainly CD4^+^ and some CD8^+^ T cells) predominated in mild lesions and B lymphocytes in severe lesions (9). Notably, we found abundant plasma cells in mild lesions, and M2 macrophages were positively associated with disease severity. Auto-antigen-specific B cells and plasma cells were thought to be related to focal fatty infiltration and promoting inflammation (46). Furthermore, the alternative activation of the M2 phenotype has been associated with severe immunopathological lesions of pSS (8). Using computational algorithms, the results also confirmed that four mitochondria-related DEGs (*CD38*, *CMPK2*, *TBC1D9*, and *PYCR1*) and immune cells are closely related. The current study advances our understanding of the linkage between mitochondria and immune cells in patients with pSS.

Mitochondria, as the primary energy-generating system, participate in a variety of biological processes, including metabolism and immune response (47, 48). Remodeling of mitochondrial content is a dynamic process with constant fission and fusion mediated by a series of conserved proteins. Mitochondrial dynamics modulate not only the mitochondrial morphology and distribution but also the cell function and fate (48, 49). In this study, we found that the gene expression relating to fission (*Fis1*, *DRP1*, *MFF*) and fusion (*MFN1*, *MFN2*, *OPA1*) was downregulated in pSS samples, consistent with the results from the public validation database. Interestingly, these genes together with mitochondria-related DEGs altered with lymphocytic infiltration in salivary glands. Another important finding from our study is that genes in the respiratory chain complexes mainly decreased in pSS associated with the degree of immune cell infiltration in salivary glands determined by various computer-aided algorithms. Meanwhile, there was a clear negative correlation of respiratory chain complex genes with *CD38* and *TBC1D9* expression levels and a positive correlation with the *PYCR1* expression level in pSS. Altered mitochondrial dynamics seem to be a potential mechanism leading to impaired mitochondrial function critical to pSS pathogenesis. Biological energy conversion in mitochondria is performed by five inner mitochondrial membrane protein complexes (electron transport complexes I–V) and two main electron carriers: soluble cytochrome c and ubiquinone Q. Once the cell is damaged, molecules carried by mitochondria (e.g., cytochrome c) are released into inappropriate compartments where they serve as DAMPs in turn recognized by immune cells (50). If released into the cytoplasm from mitochondria, cytochrome c triggers a caspase activation cascade (51) and initiates apoptosis *via* inducing Apaf-1/caspase-9 complex formation (52). According to the histological data, in healthy salivary glands, cytochrome c was strongly expressed in ductal epithelial cells, which have an abundance of mitochondria. As the lesions worsen, cytochrome c expression decreased in damaged ductal cells, whereas the expression of Bcl-2 remained strong. Meanwhile, cytochrome c protein was observed in the interstitial region infiltrated by large-scale lymphocytes. Moreover, integrative transcriptomic analysis of publicly available RNA-seq data confirmed that *Bcl-2*, *BAX*, and *caspase3* expression increased significantly in the pSS-high-infiltration group. From the above findings, we speculate that epithelial cell damage increased lymphocyte infiltration, and strategies to circumvent apoptosis or regulate cellular proliferation create a vicious cycle driving pSS pathogenesis (**Figure 6C**).

Mitochondria integrate cellular physiology, multiple signaling pathways, and cell metabolism. Previous studies have shown that different immune cells use different metabolic programs to perform their functions (49). During an immune response in pSS development, increased immune cells transit from metabolic quiescence to activation. Thus, mitochondrial metabolism can have a tremendous impact on immune cell fate and function (53). In the current study, we found that *CD38*, *CMPK2*, *TBC1D9*, and *PYCR1* are particularly important across many metabolic pathways, which also suggest substantial cross talk and potential overlap. According to previous reports, *CD38* is expressed mostly in immune cells and accumulates in inflamed tissues (54). *CMPK2* is a mitochondrial nucleotide monophosphate kinase and controls mitochondrial DNA synthesis (42), while *PYCR1* plays an important role in proline biosynthesis. Based on our results, these hub genes were found to be closely related to the mitochondrial metabolic pathway in gluconeogenesis, TCA cycle, and pyruvate/ketone/lipid/amino acid metabolism in pSS. Immune cells need appropriate levels of ATP to undertake their specific functions. Usually, activated immune cells change their metabolic state by utilizing aerobic glycolysis. Intriguingly, amino acid metabolism, especially glutamine metabolism, is also reported to be critical for immune cell development and mitochondrial immune functions (49). How alterations in metabolism affect immune responses have emerged as a potential new field in autoimmune disease.

Of note, there were still a few limitations to this study. First, the sample size of patients with pSS was small, and future studies in a larger cohort are necessary to confirm our findings. Second, although validated by transcriptomic analysis and our clinical data, further functional and validated studies are warranted to expand our results to clinical utility. Third, salivary glands contain a heterogeneous population of cells, including glandular epithelial cells, myoepithelial cells, fibroblasts, vascular cells, and immune cells, and the resulting gene expression profile of a pooled population of salivary gland cells therefore provides only an ensemble average of the cell types present. With the development of high-throughput sequencing technologies, future research in this area might include an integrated multi-omics approach based on single-cell RNA sequencing transcriptomics, proteomics, and metabolomics.

## Conclusion

We identified four hub mitochondria-related genes (*CD38*, *CMPK2*, *TBC1D9*, *PYCR1*) as a potential link between mitochondria and the immune microenvironment. Our results highlight the significance of both reduced mitochondrial dynamics and impaired respiratory chain status on pSS development. This strengthens the role of mitochondria as mediators of cellular differentiation, apoptosis, and inflammation carriers, and not as the powerhouse through OXPHOS. As pSS is an autoimmune, chronic inflammatory disease characterized by excessive lymphocytic infiltration of the exocrine gland, mitochondrial dysfunction has been proposed to contribute to pSS pathogenesis. However, many details of the immuno-metabolic mechanism orchestrated by mitochondria in pSS are still unknown. Future studies will need to investigate the different roles of mitochondria in diverse immune cells. Thus, our study provides novel insights for modulating mitochondria in the immune microenvironment for the clinical management of pSS.

## Data Availability Statement

The original contributions presented in the study are included in the article/**Supplementary Material**. Further inquiries can be directed to the corresponding authors.

## Ethics Statement

The studies involving human participants were reviewed and approved by the Ethics Committee of Ruijin Hospital, Shanghai Jiao Tong University School of Medicine. The patients/participants provided their written informed consent to participate in this study.

## Author Contributions

LJ and NL designed the overall research strategy and wrote the manuscript. YL, JH, YW, JY, HF, DL, YY, and LL performed the experiments. YG, HX, and WH analyzed the data. All authors contributed to the article and approved the submitted version.

## Funding

This work was supported by the National Natural Science Foundation of China (NSFC No. 81900975)

## Conflict of Interest

The authors declare that the research was conducted in the absence of any commercial or financial relationships that could be construed as a potential conflict of interest.

## Publisher’s Note

All claims expressed in this article are solely those of the authors and do not necessarily represent those of their affiliated organizations, or those of the publisher, the editors and the reviewers. Any product that may be evaluated in this article, or claim that may be made by its manufacturer, is not guaranteed or endorsed by the publisher.
