# Supplementary figures and images for: A Link Between Mitochondrial Dysfunction and the Immune Microenvironment of Salivary Glands in Primary Sjogren’s Syndrome
Source: Front Immunol. 2022 Mar 14;13:845209. doi: 10.3389/fimmu.2022.845209 (PMC8964148; doi:10.3389/fimmu.2022.845209)

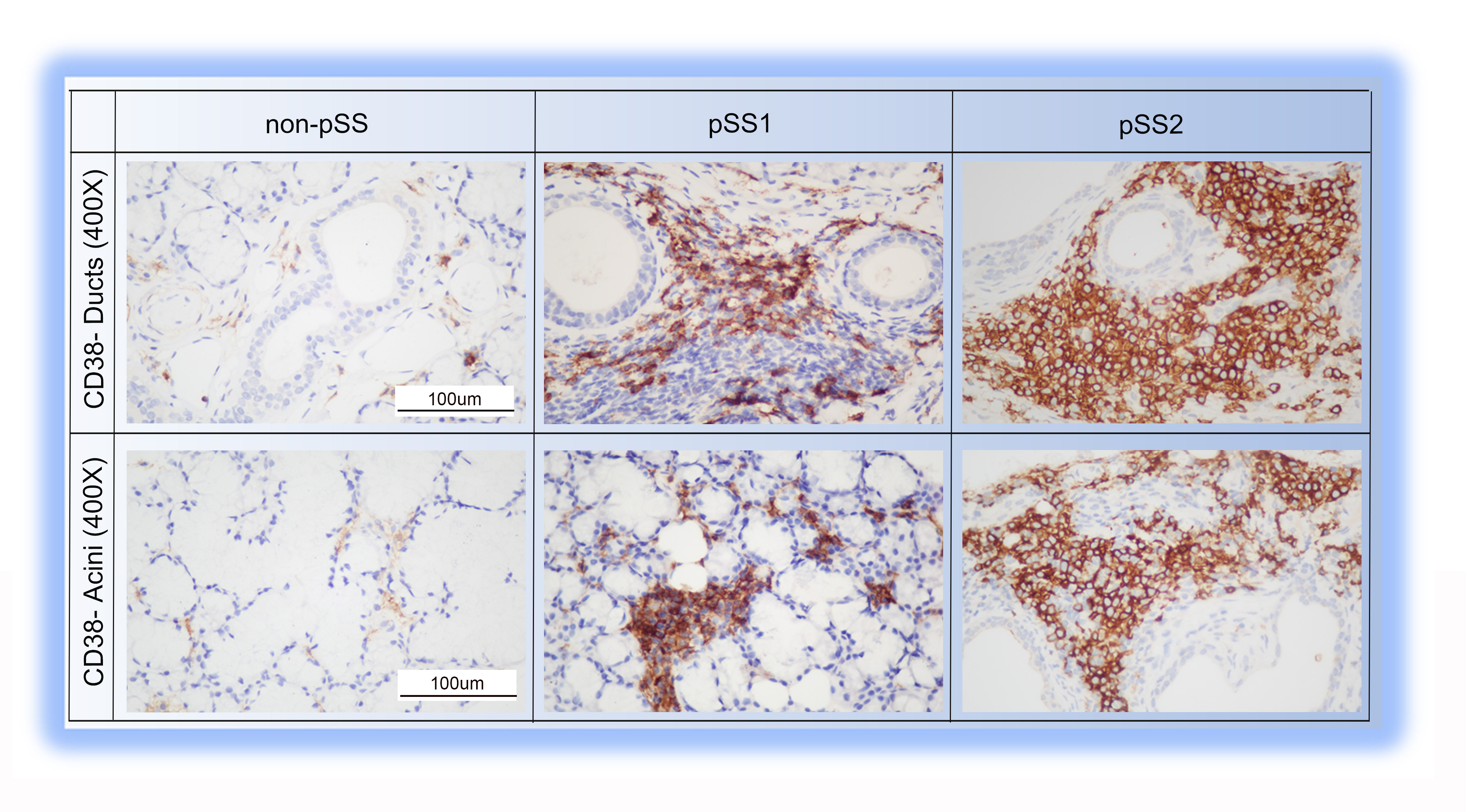

Supplement: Supplementary file 1 [file Image_1.jpeg]
