# Supplementary material for: A Link Between Mitochondrial Dysfunction and the Immune Microenvironment of Salivary Glands in Primary Sjogren’s Syndrome
Source: Front Immunol. 2022 Mar 14;13:845209. doi: 10.3389/fimmu.2022.845209 (PMC8964148; doi:10.3389/fimmu.2022.845209)
Supplement: Supplementary file 3 [file DataSheet_1.docx]

Table S1 Clinical characteristics of pSS patients included in this study.

| **Group** | **ID number** | **GENDER** | **Age**  **(years)** | **ANA** | **Anti-SSA** | **Anti-SSB** | **Anti-RO52** | **Dry mouth** | **Dry eyes** |
| --- | --- | --- | --- | --- | --- | --- | --- | --- | --- |
| **pSS1** | **N2021-182** | F | **28** | 1:640 | ++ | - | ++ | + | - |
|  | **N2021-224** | M | **21** | 1:80 | + | - | - | - | - |
|  | **N2021-227** | F | **29** | 1:80 | + | - | - | - | - |
|  | **N2021-221** | F | **68** | 1:320 | + | + | + | + | + |
|  | **N2021-134** | F | **40** | 1:80 | - | - | + | + | + |
|  | **N2021-150** | F | **57** | 1:160 | ++ | + | ++ | + | - |
|  | **N2021-226** | F | **54** | 1:80 | + | - | ++ | + | + |
|  | **N2021-123** | F | **51** | 1:80 | ++ | + | ++ | + | + |
|  | **N2021-162** | F | **52** | 1:80 | + | - | + | + | + |
|  | **N2021-210** | F | **50** | 1:320 | - | - | + | + | + |
|  | **N2021-173** | M | **60** | 1:80 | + | - | - | + | - |
|  | **N2021-235** | F | **18** | 1:320 | ++ | + | ++ | - | - |
|  | **N2021-213** | F | **25** | 1:640 | ++ | ++ | ++ | + | + |
|  | **N2021-217** | F | **32** | - | ++ | - | + | - | + |
|  | **N2021-175** | F | **41** | 1:80 | ++ | - | - | - | + |
|  | **N2021-136** | F | **30** | 1:80 | ++ | - | - | + | + |
|  | **N2021-207** | F | **55** | - | + | - | - | + | + |
|  | **N2021-211** | M | **64** | 1:160 | ++ | - | - | + | + |
|  | **N2021-212** | F | **50** | 1:160 | ++ | - | + | + | + |
|  | **N2021-216** | F | **47** | 1:80 | ++ | - | + | - | - |
|  | **N2021-189** | F | **62** | 1:80 | - | - | + | + | + |
|  | **N2021-196** | F | **34** | 1:1280 | ++ | ++ | ++ | + | + |
|  | **N2021-203** | F | **37** | 1:160 | ++ | - | ++ | + | + |
|  | **N2021-214** | M | **29** | 1:80 | ++ | - | - | + | - |
| **pSS2** | **N2021-172** | F | **42** | 1:160 | ++ | + | ++ | + | - |
|  | **N2021-178** | F | **46** | 1:80 | ++ | - | + | + | + |
|  | **N2021-139** | F | **54** | 1:160 | ++ | - | ++ | + | + |
|  | **N2021-235** | F | **18** | 1:320 | ++ | + | ++ | - | - |
|  | **N2021-180** | F | **47** | 1:1280 | ++ | ++ | + | + | + |
|  | **N2021-138** | F | **34** | 1:640 | ++ | - | ++ | + | + |
|  | **N2021-181** | F | **78** | 1:160 | ++ | - | ++- | + | + |
|  | **N2021-179** | F | **48** | 1:160 | ++ | - | ++ | + | + |
|  | **N2021-231** | F | **36** | 1:640 | ++ | - | ++ | + | + |
|  | **N2021-167** | F | **31** | 1:160 | ++ | - | - | + | + |
|  | **N2021-228** | F | **71** | 1:1280 | - | - | ++ | + | + |
|  | **N2021-229** | F | **26** | 1:80 | ++ | + | ++ | + | + |
|  | **N2021-230** | F | **61** | 1:80 | + | - | - | + | + |
|  | **N2021-265** | F | **45** | 1:640 | ++ | + | +++ | + | + |
|  | **N2021-128** | F | **25** | 1:320 | ++ | - | - | + | + |
|  | **N2021-209** | F | **57** | 1:640 | + | + | + | + | + |
|  | **N2021-126** | F | **67** | 1:640 | ++ | - | ++ | + | + |
|  | **N2021-202** | F | **41** | - | + | ++ | + | + | + |
|  | **N2021-241** | F | **48** | 1:80 | ++ | ++ | ++ | + | + |
|  | **N2021-248** | F | **55** | 1:80 | ++ | ++ | ++ | + | + |
|  | **N2021-253** | F | **51** | 1:80 | + | - | - | + | + |
|  | **N2021-268** | F | **30** | 1:160 | ++ | - | - | + | + |
|  | **N2021-260** | F | **40** | 1:1280 | - | - | + | + | + |
|  | **N2021-262** | F | **25** | 1:1280 | ++ | - | ++ | - | + |

Table S2 Summary of the forward and reverse primers of hub genes used in real-time PCR

| **Gene** | **Forward primers** | **Reverse primers** |
| --- | --- | --- |
| *CD38* | ATCCTCGTCGTGGTGCTC | TGATAGTCTTCTTCAGTAATGTTGC |
| *CMPK2* | CTCTTAAAGTCACCACCCTCTTG | GCCAGTACCTGTCTACAATCAC |
| *IFIT3* | AAGGAGAGCAGTTTGTTGAAGAAG | GGTGATAGAGGTAGCCATTGTTTG |
| *LAP3* | GCGGTGCTTGGTCTCTATG | AATCTGGTTGGCGTCATCTC |
| *PYCR1* | TGGAAGAGGACCTGATTGATGC | GCCTGGGTGCTGTTCTGAG |
| *SERHL2* | GCTGACCTACAAGCGGAGAG | CCACCTTCGTGGTTCCTCTT |
| *TBC1D9* | GCAGGAAGTTACAACAGTTCAGATG | GGAGACCGCCGCCGATAC |
| *XAF1*  *FIS1*  *DRP1*  *MFF*  *MFN1*  *MFN2*  *OPA1* | CGGTTCCTGGTCCTGTGTC  GTCCAAGAGCACGCAGTTTG  CTGCCTCAAATCGTCGTAGTG  ACTGAAGGCATTAGTCAGCGA  TGGCTAAGAAGGCGATTACTGC  CTCTCGATGCAACTCTATCGTC  TGTGAGGTCTGCCAGTCTTTA | CGCTCCTGGCACTCATTGG  ATGCCTTTACGGATGTCATCATT  GAGGTCTCCGGGTGACAATTC  TCCTGCTACAACAATCCTCTCC  TCTCCGAGATAGCACCTCACC  TCCTGTACGTGTCTTCAAGGAA  TGTCCTTAATTGGGGTCGTTG |

Table S3 GO-BP、GO-MF、GO-CC analyses of Up- and Down-regulated DEGs

| ID | Description | p.adjust | Gene ID | Count |
| --- | --- | --- | --- | --- |
| **GO-BP (Up-regulated DEGs)** | | | | |
| GO:0009141 | nucleoside triphosphate metabolic process | 1.48E-40 | CMPK2/TYMS/CDK1/PDE12/PARP1/HKDC1/TAZ/ATP5MC2/COX6A1/COX8A/NDUFA7/COX7C/COX6B1/NDUFB4/NDUFA2/UQCRH/ATP5MF/UQCR10/COX7A2/NDUFA13/ATP5MG/ATP5MC1/NDUFA11/NDUFS8/UQCR11/NDUFB11/NME4/COX5B/NDUFS5/ATP5F1E/NDUFA12/ATP5PD/ATP5F1EP2/NDUFA4/UQCRQ/NDUFA1/NDUFS6/COX7B/NDUFB3/CHCHD10/NDUFA6/COX6C/NDUFC1/ATP5ME/NDUFB1/ATP5PF/UQCC2/NDUFB2/UQCRB/NDUFB7/NDUFA3 | 51 |
| GO:0009199 | Ribonucleoside triphosphate metabolic process | 1.22E-39 | CDK1/PDE12/PARP1/HKDC1/TAZ/ATP5MC2/COX6A1/COX8A/NDUFA7/COX7C/COX6B1/NDUFB4/NDUFA2/UQCRH/ATP5MF/UQCR10/COX7A2/NDUFA13/ATP5MG/ATP5MC1/NDUFA11/NDUFS8/UQCR11/NDUFB11/NME4/COX5B/NDUFS5/ATP5F1E/NDUFA12/ATP5PD/ATP5F1EP2/NDUFA4/UQCRQ/NDUFA1/NDUFS6/COX7B/NDUFB3/CHCHD10/NDUFA6/COX6C/NDUFC1/ATP5ME/NDUFB1/ATP5PF/UQCC2/NDUFB2/UQCRB/NDUFB7/NDUFA3 | 49 |
| GO:0046034 | ATP metabolic process | 1.81E-40 | CDK1/PDE12/PARP1/HKDC1/TAZ/ATP5MC2/COX6A1/COX8A/NDUFA7/COX7C/COX6B1/NDUFB4/NDUFA2/UQCRH/ATP5MF/UQCR10/COX7A2/NDUFA13/ATP5MG/ATP5MC1/NDUFA11/NDUFS8/UQCR11/NDUFB11/COX5B/NDUFS5/ATP5F1E/NDUFA12/ATP5PD/ATP5F1EP2/NDUFA4/UQCRQ/NDUFA1/NDUFS6/COX7B/NDUFB3/CHCHD10/NDUFA6/COX6C/NDUFC1/ATP5ME/NDUFB1/ATP5PF/UQCC2/NDUFB2/UQCRB/NDUFB7/NDUFA3 | 48 |
| GO:0006119 | oxidative phosphorylation | 9.33E-52 | CDK1/PDE12/TAZ/ATP5MC2/COX6A1/COX8A/NDUFA7/COX7C/COX6B1/NDUFB4/NDUFA2/UQCRH/ATP5MF/UQCR10/COX7A2/NDUFA13/ATP5MG/ATP5MC1/NDUFA11/NDUFS8/UQCR11/NDUFB11/COX5B/NDUFS5/ATP5F1E/NDUFA12/ATP5PD/NDUFA4/UQCRQ/NDUFA1/NDUFS6/COX7B/NDUFB3/CHCHD10/NDUFA6/COX6C/NDUFC1/ATP5ME/NDUFB1/ATP5PF/UQCC2/NDUFB2/UQCRB/NDUFB7/NDUFA3 | 45 |
| GO:0022900 | electron transport chain | 1.51E-36 | CDK1/ME2/NOX4/TAZ/COX6A1/COX8A/NDUFA7/COX7C/COX6B1/NDUFB4/NDUFA2/UQCRH/UQCR10/COX7A2/NDUFA13/CYB5A/NDUFA11/NDUFS8/UQCR11/NDUFB11/COX5B/NDUFS5/NDUFA12/NDUFA4/UQCRQ/NDUFA1/GLRX5/NDUFS6/COX7B/NDUFB3/NDUFA6/COX6C/NDUFC1/NDUFB1/NDUFB2/UQCRB/NDUFB7/NDUFA3 | 38 |
| GO:0015980 | energy derivation by oxidation of organic compounds | 4.85E-28 | CDK1/LYRM7/ME2/TAZ/COX6A1/COX8A/NDUFA7/COX7C/COX6B1/NDUFB4/NDUFA2/UQCRH/UQCR10/NDUFA13/NDUFA11/NDUFS8/UQCR11/NDUFB11/COX5B/NDUFS5/NDUFA12/NDUFA4/UQCRQ/BLOC1S1/NDUFA1/NDUFS6/COX7B/NDUFB3/NDUFA6/COX6C/NDUFC1/NDUFB1/NDUFB2/UQCRB/NDUFB7/NDUFA3/ACSM1 | 37 |
| GO:0033108 | mitochondrial respiratory chain complex assembly | 2.20E-36 | SDHAF3/LYRM7/TAZ/NDUFA7/NDUFB4/NDUFA2/NDUFAF3/UQCR10/NDUFA13/COX14/COA3/NDUFA11/NDUFS8/NDUFB11/NDUFS5/NDUFA12/COX17/NDUFA1/NDUFAF8/NDUFS6/NDUFB3/NDUFA6/NDUFC1/PET100/NDUFB1/UQCC2/NDUFB2/UQCRB/NDUFB7/COX16/NDUFA3 | 31 |
| GO:0045047 | protein targeting to ER | 4.37E-24 | RPS9/RPL30/RPL23/RPS19/RPL36/RPLP2/RPL32/RPL18A/RPS15A/RPL35A/RPL31/RPS16/RPL18/RPS17/RPS13/RPL34/RPL13/RPS14/RPL10A/RPS10/RPS3/RPS8/RPL8/RPL6/RPLP0 | 25 |
| GO:0006614 | SRP-dependent cotranslational protein targeting to membrane | 2.23E-25 | RPS9/RPL30/RPL23/RPS19/RPL36/RPLP2/RPL32/RPL18A/RPS15A/RPL35A/RPL31/RPS16/RPL18/RPS17/RPS13/RPL34/RPL13/RPS14/RPL10A/RPS10/RPS3/RPS8/RPL8/RPL6/RPLP0 | 25 |
| GO:0010257 | NADH dehydrogenase complex assembly | 1.62E-24 | TAZ/NDUFA7/NDUFB4/NDUFA2/NDUFAF3/NDUFA13/NDUFA11/NDUFS8/NDUFB11/NDUFS5/NDUFA12/NDUFA1/NDUFAF8/NDUFS6/NDUFB3/NDUFA6/NDUFC1/NDUFB1/NDUFB2/NDUFB7/NDUFA3 | 21 |
| **GO-BP (Down-regulated DEGs)** | | | | |
| GO:0044282 | small molecule catabolic process | 2.39E-06 | GPT2/PLIN5/PRODH2/MECR/ALDH1L1/ACADVL/ACACB/HMGCL/MPST/TPI1/GLYCTK/DLST/ETFB/ACADS/CRAT/IMPA2/CYP27A1 | 17 |
| GO:0006732 | coenzyme metabolic process | 8.63E-06 | COASY/OGDHL/COQ9/GPD1/ALDH1L1/ACACB/HMGCL/PDK2/COQ3/TPI1/DLST/DDIT4/PC/SLC25A1/FLAD1 | 15 |
| GO:0016053 | organic acid biosynthetic process | 0.00038523 | PYCR1/GPT2/OGDHL/MECR/GPD1/ACADVL/ACACB/PTGES2/GPX4/TPI1/DDIT4/ASS1/CYP27A1 | 13 |
| GO:0046394 | carboxylic acid biosynthetic process | 0.00038523 | PYCR1/GPT2/OGDHL/MECR/GPD1/ACADVL/ACACB/PTGES2/GPX4/TPI1/DDIT4/ASS1/CYP27A1 | 13 |
| GO:0006520 | cellular amino acid metabolic process | 8.02E-05 | CKMT2/PYCR1/P4HB/GPT2/PRODH2/VARS2/NQO1/HMGCL/SARS2/COMT/MPST/DLST/ASS1 | 13 |
| GO:0051188 | cofactor biosynthetic process | 0.000113323 | COASY/OGDHL/COQ9/GPD1/ACACB/PDK2/COQ3/TPI1/DDIT4/SLC25A1/SLC25A39/FLAD1 | 12 |
| GO:0006839 | mitochondrial transport | 9.37E-05 | SLC25A29/TIMM22/SLC25A10/ALKBH7/ACACB/SLC25A37/IMMP2L/TOMM40/BAX/SLC25A1/RHOT2 | 11 |
| GO:0022900 | electron transport chain | 6.62E-05 | COQ9/GPD1/COX7A1/NQO1/PTGES2/IMMP2L/ETFB/CYCS/NDUFA4L2/TXNRD2 | 10 |
| GO:0045333 | cellular respiration | 0.001662276 | OGDHL/COQ9/GPD1/ME3/IMMP2L/DLST/ETFB/CYCS | 8 |
| GO:0006635 | fatty acid beta-oxidation | 7.67E-05 | PLIN5/MECR/ACADVL/ACACB/ETFB/ACADS/CRAT | 7 |
| **GO-MF (Up-regulated DEGs)** | | | | |
| GO:0003735 | structural constituent of ribosome | 1.02E-38 | MRPS31/RPS9/RPL30/RPL23/RPS19/RPL36/RPLP2/RPL32/RPL18A/RPS15A/RPL35A/RPL31/RPS16/RPL18/RPS17/RPS13/RPL34/RPL13/NDUFA7/RPS14/RPL10A/RPS10/MRPL36/MRPS21/RPS3/RPS8/RPL8/MRPS24/MRPL33/RPL6/MRPL51/MRPS36/MRPS33/RPLP0/MRPL14/MRPS18C/MRPL52/MRPL21/MRPL54/MRPL57/MRPL41 | 41 |
| GO:0009055 | electron transfer activity | 2.48E-17 | ME2/NOX4/COX6A1/COX8A/COX7C/COX6B1/UQCRH/UQCR10/COX7A2/CYB5A/UQCR11/COX5B/NDUFA12/NDUFA4/UQCRQ/GLRX5/NDUFS6/COX7B/COX6C/UQCRB | 20 |
| GO:0016651 | oxidoreductase activity, acting on NAD(P)H | 1.33E-16 | NOX4/CRYZ/NDUFA7/NDUFB4/NDUFA2/NDUFA13/NDUFS8/NDUFS5/NDUFA12/NDUFA4/NDUFA1/NDUFS6/NDUFB3/NDUFA6/NDUFC1/NDUFB1/NDUFB2/NDUFB7/NDUFA3 | 19 |
| GO:0015077 | monovalent inorganic cation transmembrane transporter activity | 2.63E-06 | ATP5MC2/COX6A1/COX8A/COX7C/COX6B1/COX7A2/ATP5MG/ATP5MC1/CYB5A/COX5B/ATP5F1E/ATP5PD/ATP5F1EP2/NDUFA4/COX7B/COX6C/ATP5ME/ATP5PF | 18 |
| GO:0015078 | proton transmembrane transporter activity | 1.39E-13 | ATP5MC2/COX6A1/COX8A/COX7C/COX6B1/COX7A2/ATP5MG/ATP5MC1/CYB5A/COX5B/ATP5F1E/ATP5PD/ATP5F1EP2/NDUFA4/COX7B/COX6C/ATP5ME/ATP5PF | 18 |
| GO:0016655 | oxidoreductase activity, acting on NAD(P)H, quinone or similar compound as acceptor | 5.03E-20 | CRYZ/NDUFA7/NDUFB4/NDUFA2/NDUFA13/NDUFS8/NDUFS5/NDUFA12/NDUFA4/NDUFA1/NDUFS6/NDUFB3/NDUFA6/NDUFC1/NDUFB1/NDUFB2/NDUFB7/NDUFA3 | 18 |
| GO:0004129 | cytochrome-c oxidase activity | 4.70E-12 | COX6A1/COX8A/COX7C/COX6B1/COX7A2/CYB5A/COX5B/NDUFA4/COX7B/COX6C | 10 |
| GO:0019843 | rRNA binding | 5.98E-06 | RPS9/RPL23/RPS13/RPS14/RPS3/RPL8/RPLP0/MRPS18C | 8 |
| GO:0044769 | ATPase activity, coupled to transmembrane movement of ions, rotational mechanism | 3.45E-05 | ATP5MC2/ATP5MG/ATP5MC1/ATP5F1E/ATP5PD/ATP5F1EP2 | 6 |
| GO:0046933 | proton-transporting ATP synthase activity, rotational mechanism | 5.63E-07 | ATP5MC2/ATP5MG/ATP5MC1/ATP5F1E/ATP5PD/ATP5F1EP2 | 6 |
| **GO-MF (Down-regulated DEGs)** | | | | |
| GO:0050662 | coenzyme binding | 0.000115256 | GPT2/OGDHL/PRODH2/GPD1/ACADVL/ACACB/HMGCL/ME3/ALKBH3/PC/ACADS/TXNRD2 | 12 |
| GO:0033218 | amide binding | 0.019743441 | TIMM22/ACADVL/ACACB/HMGCL/PTGES2/CRYAB/PC/AP2B1/AP2M1 | 9 |
| GO:1901681 | sulfur compound binding | 0.031916695 | OGDHL/ACADVL/ACACB/HMGCL/PTGES2/PC/ANXA6 | 7 |
| GO:0016853 | isomerase activity | 0.007273531 | P4HB/FKBP8/PTGES2/PUSL1/TPI1/FKBP10/FKBP4 | 7 |
| GO:0009055 | electron transfer activity | 0.001328639 | COX7A1/NQO1/PTGES2/ETFB/CYCS/NDUFA4L2/TXNRD2 | 7 |
| GO:0016874 | ligase activity | 0.019743441 | VARS2/ACACB/SLC27A1/SARS2/PC/ASS1 | 6 |
| GO:0016614 | oxidoreductase activity, acting on CH-OH group of donors | 0.035553278 | GPD1/L2HGDH/DHRS2/ME3/RDH13 | 5 |
| GO:0016667 | oxidoreductase activity, acting on a sulfur group of donors | 0.019743441 | P4HB/PTGES2/CCS/TXNRD2 | 4 |
| GO:0015036 | disulfide oxidoreductase activity | 0.009298148 | P4HB/PTGES2/CCS/TXNRD2 | 4 |
| GO:0008171 | O-methyltransferase activity | 0.019743441 | COMTD1/COMT/COQ3 | 3 |
| **GO-CC (Up-regulated DEGs)** | | | | |
| GO:0005743 | mitochondrial inner membrane | 3.13E-79 | IFI6/TYMS/MCUB/SLC25A32/STAR/MRPS31/TAZ/ATP5MC2/COX6A1/PAM16/HIGD2A/COX8A/NDUFA7/COX7C/MRPL53/COX6B1/MRPL36/NDUFB4/NDUFA2/UQCRH/MRPS21/ATP5MF/NDUFAF3/RPS3/UQCR10/COX7A2/NDUFA13/ATP5MG/ATP5MC1/ROMO1/COA3/NDUFA11/NDUFS8/UQCR11/NDUFB11/NME4/TIMM9/MRPS24/COX5B/NDUFS5/ATP5MD/ATP5F1E/MRPL33/NDUFA12/MRPL51/MRPS36/ATP5PD/SMDT1/ATP5F1EP2/CHCHD1/ATP5MPL/MRPS33/NDUFA4/UQCRQ/MRPL14/NDUFA1/MRPS18C/MRPL52/MRPL21/NDUFS6/COX7B/TMEM126A/MRPL54/NDUFB3/CHCHD10/MRPL57/NDUFA6/COX6C/MRPL41/NDUFC1/PET100/ATP5ME/NDUFB1/ATP5PF/UQCC2/IMMP1L/NDUFB2/UQCRB/NDUFB7/COX16/TIMM8B/NDUFA3/AIFM3 | 83 |
| GO:0098798 | mitochondrial protein complex | 5.50E-73 | MCUB/PDK1/MRPS31/ATP5MC2/COX6A1/PAM16/NDUFA7/MRPL53/MRPL36/NDUFB4/TOMM7/NDUFA2/UQCRH/MRPS21/ATP5MF/UQCR10/NDUFA13/ATP5MG/TOMM6/ATP5MC1/ROMO1/COA3/NDUFA11/NDUFS8/NDUFB11/TIMM9/MRPS24/NDUFS5/ATP5MD/ATP5F1E/MRPL33/NDUFA12/MRPL51/MRPS36/ATP5PD/SMDT1/ATP5F1EP2/CHCHD1/ATP5MPL/MRPS33/NDUFA4/UQCRQ/MRPL14/NDUFA1/TOMM5/MRPS18C/MRPL52/MRPL21/NDUFS6/MRPL54/NDUFB3/CHCHD10/MRPL57/NDUFA6/MRPL41/NDUFC1/ATP5ME/NDUFB1/ATP5PF/IMMP1L/NDUFB2/UQCRB/NDUFB7/TIMM8B/POLRMT/NDUFA3 | 66 |
| GO:0005840 | ribosome | 1.02E-38 | MRPS31/RPS9/RPL30/RPL23/RPS19/RPL36/RPLP2/RPL32/RPL18A/RPS15A/RPL35A/RPL31/RPS16/RPL18/RPS17/RPS13/RPL34/RPL13/NDUFA7/RPS14/RPL10A/MRPL53/RPS10/MRPL36/MRPS21/RPS3/RPS8/RACK1/RPL8/MRPS24/MRPL33/RPL6/MRPL51/MRPS36/CHCHD1/MRPS33/RPLP0/MRPL14/MRPS18C/MRPL52/MRPL21/MRPL54/MRPL57/MRPL41 | 44 |
| GO:0005759 | mitochondrial matrix | 2.02E-27 | TYMS/CDK1/SDHAF3/TRNT1/LYRM7/PDE12/ME2/PDK1/MRPS31/PAM16/NDUFA7/MRPL53/MRPL36/MRPS21/RPS3/ALKBH7/NDUFS8/NME4/MRPS24/PIN4/ATP5F1E/MRPL33/GPX1/MRPL51/MRPS36/SMDT1/CHCHD1/MRPS33/MRPL14/BLOC1S1/MRPS18C/GLRX5/MRPL52/MRPL21/MRPL54/MRPL57/ECI1/MRPL41/UQCC2/ETHE1/GLYAT/POLRMT/ACSM1 | 43 |
| GO:0070469 | respiratory chain | 1.67E-36 | COX6A1/HIGD2A/COX8A/NDUFA7/COX6B1/NDUFB4/NDUFA2/UQCRH/UQCR10/COX7A2/NDUFA13/COA3/NDUFA11/NDUFS8/UQCR11/NDUFB11/NDUFS5/NDUFA12/NDUFA4/UQCRQ/NDUFA1/NDUFS6/COX7B/NDUFB3/NDUFA6/NDUFC1/NDUFB1/NDUFB2/UQCRB/NDUFB7/NDUFA3 | 31 |
| GO:0044445 | cytosolic part | 3.99E-19 | RPS9/RPL30/RPL23/RPS19/RPL36/RPLP2/RPL32/RPL18A/RPS15A/RPL35A/RPL31/RPS16/RPL18/RPS17/RPS13/RPL34/RPL13/RPS14/RPL10A/RPS10/RPS3/RPS8/RACK1/RPL8/RPL6/RPLP0/BLOC1S1 | 27 |
| GO:1990204 | oxidoreductase complex | 4.82E-27 | PDK1/NOX4/NDUFA7/NDUFB4/NDUFA2/UQCRH/UQCR10/NDUFA13/NDUFA11/NDUFS8/NDUFB11/NDUFS5/NDUFA12/MRPS36/NDUFA4/UQCRQ/NDUFA1/NDUFS6/NDUFB3/NDUFA6/NDUFC1/NDUFB1/NDUFB2/UQCRB/NDUFB7/NDUFA3 | 26 |
| GO:0005925 | focal adhesion | 1.10E-09 | LAP3/VIM/NOX4/HSP90B1/RPS9/RPL30/RPL23/RPS19/RPLP2/RPL31/RPS16/RPL18/RPS17/RPS13/RPS14/RPL10A/RPS10/RPS3/RPS8/RPL8/RPL6/RPLP0 | 22 |
| GO:0030964 | NADH dehydrogenase complex | 1.69E-24 | NDUFA7/NDUFB4/NDUFA2/NDUFA13/NDUFA11/NDUFS8/NDUFB11/NDUFS5/NDUFA12/NDUFA4/NDUFA1/NDUFS6/NDUFB3/NDUFA6/NDUFC1/NDUFB1/NDUFB2/NDUFB7/NDUFA3 | 19 |
| GO:0005753 | mitochondrial proton-transporting ATP synthase complex | 5.52E-16 | ATP5MC2/ATP5MF/ATP5MG/ATP5MC1/ATP5MD/ATP5F1E/ATP5PD/ATP5F1EP2/ATP5MPL/ATP5ME/ATP5PF | 11 |
| **GO-CC (Down-regulated DEGs)** | | | | |
| GO:0005743 | mitochondrial inner membrane | 2.92E-18 | CKMT2/NME4/SLC25A29/PRODH2/SLC25A34/COQ9/TIMM22/SLC25A10/MRPL37/COX7A1/ACADVL/SPNS1/L2HGDH/SLC27A1/MRPL41/SLC25A37/COQ3/IMMP2L/MRPL21/EFHD1/TOMM40/SLC25A1/SLC25A39/CYCS/CRAT/NDUFA4L2/RDH13/CYP27A1 | 28 |
| GO:0005759 | mitochondrial matrix | 1.61E-17 | PYCR1/GPT2/COASY/OGDHL/NME4/MECR/MRPL37/ACADVL/ALKBH7/HMGCL/DHRS2/SARS2/PDK2/MRPL41/ME3/COQ3/MPST/MRPL21/TMLHE/DLST/PC/ATG4D/ETFB/ACADS/FLAD1/TXNRD2/CYP27A1 | 27 |
| GO:0005741 | mitochondrial outer membrane | 6.80E-06 | COASY/MAVS/ACACB/QTRT1/TOMM40/BAX/PGAM5/ASS1/RHOT2/VAMP1 | 10 |
| GO:0098798 | mitochondrial protein complex | 0.000859 | TIMM22/MRPL37/PDK2/MRPL41/IMMP2L/MRPL21/TOMM40/BAX/NDUFA4L2 | 9 |
| GO:0042579 | microbody | 0.025214 | SERHL2/MAVS/HMGCL/PXMP2/CRAT | 5 |
| GO:0005777 | peroxisome | 0.025214 | SERHL2/MAVS/HMGCL/PXMP2/CRAT | 5 |
| GO:1990204 | oxidoreductase complex | 0.014015 | P4HB/OGDHL/GPD1/PDK2/DLST | 5 |
| GO:0005762 | mitochondrial large ribosomal subunit | 0.047551 | MRPL37/MRPL41/MRPL21 | 3 |

Table S4 Gene Set Enrichment Analysis (GSEA) of KEGG pathway enrichment for CD38, CMPK2, TBC1D9, and PYCR1 high expression group versus low expression group

| **CD38** | | | | | | | | |
| --- | --- | --- | --- | --- | --- | --- | --- | --- |
| **Follow link to MSigDB** | **GS DETAILS** | **ES** | **NES** | **NOM p-val** | **FDR q-val** | **FWER p-val** | **RANK AT MAX** | **LEADING EDGE** |
| [KEGG_ANTIGEN_PROCESSING_AND_PRESENTATION](http://www.gsea-msigdb.org/gsea/msigdb/cards/KEGG_ANTIGEN_PROCESSING_AND_PRESENTATION) | [Details ...](file:///C:\..\..\2019%E5%90%B4%E6%A2%A6%E7%91%B6-paper\%E7%94%9F%E4%BF%A1%E5%88%86%E6%9E%90\%E7%BB%93%E6%9E%9C\%E7%BA%BF%E7%B2%92%E4%BD%93\GSEA_RESULTS\CD38_KEGG.Gsea.1621000389657\KEGG_ANTIGEN_PROCESSING_AND_PRESENTATION.html) | 0.72 | 1.84 | 0 | 0.002 | 0.003 | 2759 | tags=43%, list=8%, signal=47% |
| [KEGG_NATURAL_KILLER_CELL_MEDIATED_CYTOTOXICITY](http://www.gsea-msigdb.org/gsea/msigdb/cards/KEGG_NATURAL_KILLER_CELL_MEDIATED_CYTOTOXICITY) | [Details ...](file:///C:\..\..\2019%25E5%2590%25B4%25E6%25A2%25A6%25E7%2591%25B6-paper\%25E7%2594%259F%25E4%25BF%25A1%25E5%2588%2586%25E6%259E%2590\%25E7%25BB%2593%25E6%259E%259C\%25E7%25BA%25BF%25E7%25B2%2592%25E4%25BD%2593\GSEA_RESULTS\CD38_KEGG.Gsea.1621000389657\KEGG_NATURAL_KILLER_CELL_MEDIATED_CYTOTOXICITY.html) | 0.72 | 1.84 | 0 | 0.001 | 0.003 | 3130 | tags=45%, list=9%, signal=49% |
| [KEGG_PRIMARY_IMMUNODEFICIENCY](http://www.gsea-msigdb.org/gsea/msigdb/cards/KEGG_PRIMARY_IMMUNODEFICIENCY) | [Details ...](file:///C:\..\..\2019%25E5%2590%25B4%25E6%25A2%25A6%25E7%2591%25B6-paper\%25E7%2594%259F%25E4%25BF%25A1%25E5%2588%2586%25E6%259E%2590\%25E7%25BB%2593%25E6%259E%259C\%25E7%25BA%25BF%25E7%25B2%2592%25E4%25BD%2593\GSEA_RESULTS\CD38_KEGG.Gsea.1621000389657\KEGG_PRIMARY_IMMUNODEFICIENCY.html) | 0.86 | 1.76 | 0 | 0.005 | 0.019 | 2457 | tags=73%, list=7%, signal=78% |
| [KEGG_CYTOKINE_CYTOKINE_RECEPTOR_INTERACTION](http://www.gsea-msigdb.org/gsea/msigdb/cards/KEGG_CYTOKINE_CYTOKINE_RECEPTOR_INTERACTION) | [Details ...](file:///C:\..\..\2019%25E5%2590%25B4%25E6%25A2%25A6%25E7%2591%25B6-paper\%25E7%2594%259F%25E4%25BF%25A1%25E5%2588%2586%25E6%259E%2590\%25E7%25BB%2593%25E6%259E%259C\%25E7%25BA%25BF%25E7%25B2%2592%25E4%25BD%2593\GSEA_RESULTS\CD38_KEGG.Gsea.1621000389657\KEGG_CYTOKINE_CYTOKINE_RECEPTOR_INTERACTION.html) | 0.62 | 1.72 | 0.002 | 0.01 | 0.037 | 2324 | tags=33%, list=7%, signal=35% |
| [KEGG_CELL_ADHESION_MOLECULES_CAMS](http://www.gsea-msigdb.org/gsea/msigdb/cards/KEGG_CELL_ADHESION_MOLECULES_CAMS) | [Details ...](file:///C:\..\..\2019%25E5%2590%25B4%25E6%25A2%25A6%25E7%2591%25B6-paper\%25E7%2594%259F%25E4%25BF%25A1%25E5%2588%2586%25E6%259E%2590\%25E7%25BB%2593%25E6%259E%259C\%25E7%25BA%25BF%25E7%25B2%2592%25E4%25BD%2593\GSEA_RESULTS\CD38_KEGG.Gsea.1621000389657\KEGG_CELL_ADHESION_MOLECULES_CAMS.html) | 0.64 | 1.66 | 0.002 | 0.017 | 0.084 | 1962 | tags=33%, list=6%, signal=34% |
| [KEGG_T_CELL_RECEPTOR_SIGNALING_PATHWAY](http://www.gsea-msigdb.org/gsea/msigdb/cards/KEGG_T_CELL_RECEPTOR_SIGNALING_PATHWAY) | [Details ...](file:///C:\..\..\2019%E5%90%B4%E6%A2%A6%E7%91%B6-paper\%E7%94%9F%E4%BF%A1%E5%88%86%E6%9E%90\%E7%BB%93%E6%9E%9C\%E7%BA%BF%E7%B2%92%E4%BD%93\GSEA_RESULTS\CD38_KEGG.Gsea.1621000389657\KEGG_T_CELL_RECEPTOR_SIGNALING_PATHWAY.html) | 0.66 | 1.6 | 0 | 0.027 | 0.164 | 3558 | tags=41%, list=11%, signal=46% |
| [KEGG_JAK_STAT_SIGNALING_PATHWAY](http://www.gsea-msigdb.org/gsea/msigdb/cards/KEGG_JAK_STAT_SIGNALING_PATHWAY) | [Details ...](file:///C:\..\..\2019%25E5%2590%25B4%25E6%25A2%25A6%25E7%2591%25B6-paper\%25E7%2594%259F%25E4%25BF%25A1%25E5%2588%2586%25E6%259E%2590\%25E7%25BB%2593%25E6%259E%259C\%25E7%25BA%25BF%25E7%25B2%2592%25E4%25BD%2593\GSEA_RESULTS\CD38_KEGG.Gsea.1621000389657\KEGG_JAK_STAT_SIGNALING_PATHWAY.html) | 0.6 | 1.58 | 0.006 | 0.032 | 0.208 | 4611 | tags=35%, list=14%, signal=40% |
| [KEGG_B_CELL_RECEPTOR_SIGNALING_PATHWAY](http://www.gsea-msigdb.org/gsea/msigdb/cards/KEGG_B_CELL_RECEPTOR_SIGNALING_PATHWAY) | [Details ...](file:///C:\..\..\2019%25E5%2590%25B4%25E6%25A2%25A6%25E7%2591%25B6-paper\%25E7%2594%259F%25E4%25BF%25A1%25E5%2588%2586%25E6%259E%2590\%25E7%25BB%2593%25E6%259E%259C\%25E7%25BA%25BF%25E7%25B2%2592%25E4%25BD%2593\GSEA_RESULTS\CD38_KEGG.Gsea.1621000389657\KEGG_B_CELL_RECEPTOR_SIGNALING_PATHWAY.html) | 0.65 | 1.5 | 0.012 | 0.064 | 0.38 | 3558 | tags=41%, list=11%, signal=46% |
| **CMPK2** | | | | | | | | |
| **Follow link to MSigDB** | **GS DETAILS** | **ES** | **NES** | **NOM p-val** | **FDR q-val** | **FWER p-val** | **RANK AT MAX** | **LEADING EDGE** |
| [KEGG_CYTOKINE_CYTOKINE_RECEPTOR_INTERACTION](http://www.gsea-msigdb.org/gsea/msigdb/cards/KEGG_CYTOKINE_CYTOKINE_RECEPTOR_INTERACTION) | [Details ...](file:///C:\..\..\2019%25E5%2590%25B4%25E6%25A2%25A6%25E7%2591%25B6-paper\%25E7%2594%259F%25E4%25BF%25A1%25E5%2588%2586%25E6%259E%2590\%25E7%25BB%2593%25E6%259E%259C\%25E7%25BA%25BF%25E7%25B2%2592%25E4%25BD%2593\GSEA_RESULTS\CMPK2_KEGG.Gsea.1621000770222\KEGG_CYTOKINE_CYTOKINE_RECEPTOR_INTERACTION.html) | 0.64 | 1.76 | 0 | 0.011 | 0.011 | 4373 | tags=44%, list=13%, signal=51% |
| [KEGG_NATURAL_KILLER_CELL_MEDIATED_CYTOTOXICITY](http://www.gsea-msigdb.org/gsea/msigdb/cards/KEGG_NATURAL_KILLER_CELL_MEDIATED_CYTOTOXICITY) | [Details ...](file:///C:\..\..\2019%25E5%2590%25B4%25E6%25A2%25A6%25E7%2591%25B6-paper\%25E7%2594%259F%25E4%25BF%25A1%25E5%2588%2586%25E6%259E%2590\%25E7%25BB%2593%25E6%259E%259C\%25E7%25BA%25BF%25E7%25B2%2592%25E4%25BD%2593\GSEA_RESULTS\CMPK2_KEGG.Gsea.1621000770222\KEGG_NATURAL_KILLER_CELL_MEDIATED_CYTOTOXICITY.html) | 0.66 | 1.7 | 0.002 | 0.029 | 0.045 | 6259 | tags=52%, list=19%, signal=63% |
| [KEGG_PRIMARY_IMMUNODEFICIENCY](http://www.gsea-msigdb.org/gsea/msigdb/cards/KEGG_PRIMARY_IMMUNODEFICIENCY) | [Details ...](file:///C:\..\..\2019%25E5%2590%25B4%25E6%25A2%25A6%25E7%2591%25B6-paper\%25E7%2594%259F%25E4%25BF%25A1%25E5%2588%2586%25E6%259E%2590\%25E7%25BB%2593%25E6%259E%259C\%25E7%25BA%25BF%25E7%25B2%2592%25E4%25BD%2593\GSEA_RESULTS\CMPK2_KEGG.Gsea.1621000770222\KEGG_PRIMARY_IMMUNODEFICIENCY.html) | 0.82 | 1.68 | 0.002 | 0.027 | 0.066 | 2529 | tags=67%, list=8%, signal=72% |
| [KEGG_T_CELL_RECEPTOR_SIGNALING_PATHWAY](http://www.gsea-msigdb.org/gsea/msigdb/cards/KEGG_T_CELL_RECEPTOR_SIGNALING_PATHWAY) | [Details ...](file:///C:\..\..\2019%25E5%2590%25B4%25E6%25A2%25A6%25E7%2591%25B6-paper\%25E7%2594%259F%25E4%25BF%25A1%25E5%2588%2586%25E6%259E%2590\%25E7%25BB%2593%25E6%259E%259C\%25E7%25BA%25BF%25E7%25B2%2592%25E4%25BD%2593\GSEA_RESULTS\CMPK2_KEGG.Gsea.1621000770222\KEGG_T_CELL_RECEPTOR_SIGNALING_PATHWAY.html) | 0.69 | 1.67 | 0 | 0.026 | 0.074 | 6229 | tags=61%, list=19%, signal=75% |
| [KEGG_CHEMOKINE_SIGNALING_PATHWAY](http://www.gsea-msigdb.org/gsea/msigdb/cards/KEGG_CHEMOKINE_SIGNALING_PATHWAY) | [Details ...](file:///C:\..\..\2019%25E5%2590%25B4%25E6%25A2%25A6%25E7%2591%25B6-paper\%25E7%2594%259F%25E4%25BF%25A1%25E5%2588%2586%25E6%259E%2590\%25E7%25BB%2593%25E6%259E%259C\%25E7%25BA%25BF%25E7%25B2%2592%25E4%25BD%2593\GSEA_RESULTS\CMPK2_KEGG.Gsea.1621000770222\KEGG_CHEMOKINE_SIGNALING_PATHWAY.html) | 0.64 | 1.65 | 0 | 0.03 | 0.109 | 5636 | tags=48%, list=17%, signal=58% |
| [KEGG_TOLL_LIKE_RECEPTOR_SIGNALING_PATHWAY](http://www.gsea-msigdb.org/gsea/msigdb/cards/KEGG_TOLL_LIKE_RECEPTOR_SIGNALING_PATHWAY) | [Details ...](file:///C:\..\..\2019%25E5%2590%25B4%25E6%25A2%25A6%25E7%2591%25B6-paper\%25E7%2594%259F%25E4%25BF%25A1%25E5%2588%2586%25E6%259E%2590\%25E7%25BB%2593%25E6%259E%259C\%25E7%25BA%25BF%25E7%25B2%2592%25E4%25BD%2593\GSEA_RESULTS\CMPK2_KEGG.Gsea.1621000770222\KEGG_TOLL_LIKE_RECEPTOR_SIGNALING_PATHWAY.html) | 0.67 | 1.64 | 0 | 0.028 | 0.111 | 6243 | tags=55%, list=19%, signal=67% |
| [KEGG_JAK_STAT_SIGNALING_PATHWAY](http://www.gsea-msigdb.org/gsea/msigdb/cards/KEGG_JAK_STAT_SIGNALING_PATHWAY) | [Details ...](file:///C:\..\..\2019%25E5%2590%25B4%25E6%25A2%25A6%25E7%2591%25B6-paper\%25E7%2594%259F%25E4%25BF%25A1%25E5%2588%2586%25E6%259E%2590\%25E7%25BB%2593%25E6%259E%259C\%25E7%25BA%25BF%25E7%25B2%2592%25E4%25BD%2593\GSEA_RESULTS\CMPK2_KEGG.Gsea.1621000770222\KEGG_JAK_STAT_SIGNALING_PATHWAY.html) | 0.62U | 1.64 | 0 | 0.026 | 0.113 | 6028 | tags=46%, list=18%, signal=56% |
| [KEGG_B_CELL_RECEPTOR_SIGNALING_PATHWAY](http://www.gsea-msigdb.org/gsea/msigdb/cards/KEGG_B_CELL_RECEPTOR_SIGNALING_PATHWAY) | [Details ...](file:///C:\..\..\2019%25E5%2590%25B4%25E6%25A2%25A6%25E7%2591%25B6-paper\%25E7%2594%259F%25E4%25BF%25A1%25E5%2588%2586%25E6%259E%2590\%25E7%25BB%2593%25E6%259E%259C\%25E7%25BA%25BF%25E7%25B2%2592%25E4%25BD%2593\GSEA_RESULTS\CMPK2_KEGG.Gsea.1621000770222\KEGG_B_CELL_RECEPTOR_SIGNALING_PATHWAY.html) | 0.69 | 1.62 | 0 | 0.034 | 0.149 | 6229 | tags=64%, list=19%, signal=79% |
| [KEGG_VEGF_SIGNALING_PATHWAY](http://www.gsea-msigdb.org/gsea/msigdb/cards/KEGG_VEGF_SIGNALING_PATHWAY) | [Details ...](file:///C:\..\..\2019%25E5%2590%25B4%25E6%25A2%25A6%25E7%2591%25B6-paper\%25E7%2594%259F%25E4%25BF%25A1%25E5%2588%2586%25E6%259E%2590\%25E7%25BB%2593%25E6%259E%259C\%25E7%25BA%25BF%25E7%25B2%2592%25E4%25BD%2593\GSEA_RESULTS\CMPK2_KEGG.Gsea.1621000770222\KEGG_VEGF_SIGNALING_PATHWAY.html) | 0.54 | 1.42 | 0.024 | 0.134 | 0.571 | 6254 | tags=43%, list=19%, signal=53% |
| [KEGG_MAPK_SIGNALING_PATHWAY](http://www.gsea-msigdb.org/gsea/msigdb/cards/KEGG_MAPK_SIGNALING_PATHWAY) | [Details ...](file:///C:\..\..\2019%25E5%2590%25B4%25E6%25A2%25A6%25E7%2591%25B6-paper\%25E7%2594%259F%25E4%25BF%25A1%25E5%2588%2586%25E6%259E%2590\%25E7%25BB%2593%25E6%259E%259C\%25E7%25BA%25BF%25E7%25B2%2592%25E4%25BD%2593\GSEA_RESULTS\CMPK2_KEGG.Gsea.1621000770222\KEGG_MAPK_SIGNALING_PATHWAY.html) | 0.51 | 1.41 | 0.02 | 0.151 | 0.602 | 9978 | tags=56%, list=30%, signal=79% |
| [KEGG_PATHWAYS_IN_CANCER](http://www.gsea-msigdb.org/gsea/msigdb/cards/KEGG_PATHWAYS_IN_CANCER) | [Details ...](file:///C:\..\..\2019%25E5%2590%25B4%25E6%25A2%25A6%25E7%2591%25B6-paper\%25E7%2594%259F%25E4%25BF%25A1%25E5%2588%2586%25E6%259E%2590\%25E7%25BB%2593%25E6%259E%259C\%25E7%25BA%25BF%25E7%25B2%2592%25E4%25BD%2593\GSEA_RESULTS\CMPK2_KEGG.Gsea.1621000770222\KEGG_PATHWAYS_IN_CANCER.html) | 0.53 | 1.39 | 0.037 | 0.165 | 0.626 | 10892 | tags=61%, list=33%, signal=90% |
| **PRCY1** | | | | | | | | |
| **Follow link to MSigDB** | **GS DETAILS** | **ES** | **NES** | **NOM p-val** | **FDR q-val** | **FWER p-val** | **RANK AT MAX** | **LEADING EDGE** |
| [KEGG_JAK_STAT_SIGNALING_PATHWAY](http://www.gsea-msigdb.org/gsea/msigdb/cards/KEGG_JAK_STAT_SIGNALING_PATHWAY) | [Details ...](file:///C:\..\2019%25E5%2590%25B4%25E6%25A2%25A6%25E7%2591%25B6-paper\%25E7%2594%259F%25E4%25BF%25A1%25E5%2588%2586%25E6%259E%2590\%25E7%25BB%2593%25E6%259E%259C\%25E7%25BA%25BF%25E7%25B2%2592%25E4%25BD%2593\GSEA_RESULTS\DOWN_GSEA\PYCR1_KEGG.Gsea.1622039013333\KEGG_JAK_STAT_SIGNALING_PATHWAY.html) | -0.56 | -1.87 | 0 | 0.049 | 0.033 | 6248 | tags=49%, list=19%, signal=60% |
| [KEGG_CYTOKINE_CYTOKINE_RECEPTOR_INTERACTION](http://www.gsea-msigdb.org/gsea/msigdb/cards/KEGG_CYTOKINE_CYTOKINE_RECEPTOR_INTERACTION) | [Details ...](file:///C:\..\2019%25E5%2590%25B4%25E6%25A2%25A6%25E7%2591%25B6-paper\%25E7%2594%259F%25E4%25BF%25A1%25E5%2588%2586%25E6%259E%2590\%25E7%25BB%2593%25E6%259E%259C\%25E7%25BA%25BF%25E7%25B2%2592%25E4%25BD%2593\GSEA_RESULTS\DOWN_GSEA\PYCR1_KEGG.Gsea.1622039013333\KEGG_CYTOKINE_CYTOKINE_RECEPTOR_INTERACTION.html) | -0.54 | -1.8 | 0 | 0.051 | 0.093 | 6295 | tags=50%, list=19%, signal=61% |
| [KEGG_CHEMOKINE_SIGNALING_PATHWAY](http://www.gsea-msigdb.org/gsea/msigdb/cards/KEGG_CHEMOKINE_SIGNALING_PATHWAY) | [Details ...](file:///C:\..\2019%25E5%2590%25B4%25E6%25A2%25A6%25E7%2591%25B6-paper\%25E7%2594%259F%25E4%25BF%25A1%25E5%2588%2586%25E6%259E%2590\%25E7%25BB%2593%25E6%259E%259C\%25E7%25BA%25BF%25E7%25B2%2592%25E4%25BD%2593\GSEA_RESULTS\DOWN_GSEA\PYCR1_KEGG.Gsea.1622039013333\KEGG_CHEMOKINE_SIGNALING_PATHWAY.html) | -0.58 | -1.8 | 0.006 | 0.043 | 0.102 | 8289 | tags=60%, list=25%, signal=79% |
| [KEGG_TGF_BETA_SIGNALING_PATHWAY](http://www.gsea-msigdb.org/gsea/msigdb/cards/KEGG_TGF_BETA_SIGNALING_PATHWAY) | [Details ...](file:///C:\..\2019%25E5%2590%25B4%25E6%25A2%25A6%25E7%2591%25B6-paper\%25E7%2594%259F%25E4%25BF%25A1%25E5%2588%2586%25E6%259E%2590\%25E7%25BB%2593%25E6%259E%259C\%25E7%25BA%25BF%25E7%25B2%2592%25E4%25BD%2593\GSEA_RESULTS\DOWN_GSEA\PYCR1_KEGG.Gsea.1622039013333\KEGG_TGF_BETA_SIGNALING_PATHWAY.html) | -0.51 | -1.74 | 0.004 | 0.065 | 0.163 | 3493 | tags=33%, list=11%, signal=37% |
| [KEGG_PATHWAYS_IN_CANCER](http://www.gsea-msigdb.org/gsea/msigdb/cards/KEGG_PATHWAYS_IN_CANCER) | [Details ...](file:///C:\..\2019%25E5%2590%25B4%25E6%25A2%25A6%25E7%2591%25B6-paper\%25E7%2594%259F%25E4%25BF%25A1%25E5%2588%2586%25E6%259E%2590\%25E7%25BB%2593%25E6%259E%259C\%25E7%25BA%25BF%25E7%25B2%2592%25E4%25BD%2593\GSEA_RESULTS\DOWN_GSEA\PYCR1_KEGG.Gsea.1622039013333\KEGG_PATHWAYS_IN_CANCER.html) | -0.49 | -1.74 | 0 | 0.057 | 0.169 | 7116 | tags=45%, list=21%, signal=57% |
| [KEGG_ECM_RECEPTOR_INTERACTION](http://www.gsea-msigdb.org/gsea/msigdb/cards/KEGG_ECM_RECEPTOR_INTERACTION) | [Details ...](file:///C:\..\2019%25E5%2590%25B4%25E6%25A2%25A6%25E7%2591%25B6-paper\%25E7%2594%259F%25E4%25BF%25A1%25E5%2588%2586%25E6%259E%2590\%25E7%25BB%2593%25E6%259E%259C\%25E7%25BA%25BF%25E7%25B2%2592%25E4%25BD%2593\GSEA_RESULTS\DOWN_GSEA\PYCR1_KEGG.Gsea.1622039013333\KEGG_ECM_RECEPTOR_INTERACTION.html) | -0.66 | -1.68 | 0 | 0.062 | 0.266 | 6173 | tags=64%, list=19%, signal=78% |
| [KEGG_MAPK_SIGNALING_PATHWAY](http://www.gsea-msigdb.org/gsea/msigdb/cards/KEGG_MAPK_SIGNALING_PATHWAY) | [Details ...](file:///C:\..\2019%25E5%2590%25B4%25E6%25A2%25A6%25E7%2591%25B6-paper\%25E7%2594%259F%25E4%25BF%25A1%25E5%2588%2586%25E6%259E%2590\%25E7%25BB%2593%25E6%259E%259C\%25E7%25BA%25BF%25E7%25B2%2592%25E4%25BD%2593\GSEA_RESULTS\DOWN_GSEA\PYCR1_KEGG.Gsea.1622039013333\KEGG_MAPK_SIGNALING_PATHWAY.html) | -0.46 | -1.66 | 0.01 | 0.075 | 0.33 | 6042 | tags=39%, list=18%, signal=47% |
| [KEGG_CELL_ADHESION_MOLECULES_CAMS](http://www.gsea-msigdb.org/gsea/msigdb/cards/KEGG_CELL_ADHESION_MOLECULES_CAMS) | [Details ...](file:///C:\..\2019%25E5%2590%25B4%25E6%25A2%25A6%25E7%2591%25B6-paper\%25E7%2594%259F%25E4%25BF%25A1%25E5%2588%2586%25E6%259E%2590\%25E7%25BB%2593%25E6%259E%259C\%25E7%25BA%25BF%25E7%25B2%2592%25E4%25BD%2593\GSEA_RESULTS\DOWN_GSEA\PYCR1_KEGG.Gsea.1622039013333\KEGG_CELL_ADHESION_MOLECULES_CAMS.html) | -0.49 | -1.64 | 0.008 | 0.082 | 0.362 | 4613 | tags=40%, list=14%, signal=46% |
| [KEGG_T_CELL_RECEPTOR_SIGNALING_PATHWAY](http://www.gsea-msigdb.org/gsea/msigdb/cards/KEGG_T_CELL_RECEPTOR_SIGNALING_PATHWAY) | [Details ...](file:///C:\..\2019%25E5%2590%25B4%25E6%25A2%25A6%25E7%2591%25B6-paper\%25E7%2594%259F%25E4%25BF%25A1%25E5%2588%2586%25E6%259E%2590\%25E7%25BB%2593%25E6%259E%259C\%25E7%25BA%25BF%25E7%25B2%2592%25E4%25BD%2593\GSEA_RESULTS\DOWN_GSEA\PYCR1_KEGG.Gsea.1622039013333\KEGG_T_CELL_RECEPTOR_SIGNALING_PATHWAY.html) | -0.54 | -1.63 | 0.013 | 0.079 | 0.397 | 7254 | tags=59%, list=22%, signal=75% |
| [KEGG_FOCAL_ADHESION](http://www.gsea-msigdb.org/gsea/msigdb/cards/KEGG_FOCAL_ADHESION) | [Details ...](file:///C:\..\2019%25E5%2590%25B4%25E6%25A2%25A6%25E7%2591%25B6-paper\%25E7%2594%259F%25E4%25BF%25A1%25E5%2588%2586%25E6%259E%2590\%25E7%25BB%2593%25E6%259E%259C\%25E7%25BA%25BF%25E7%25B2%2592%25E4%25BD%2593\GSEA_RESULTS\DOWN_GSEA\PYCR1_KEGG.Gsea.1622039013333\KEGG_FOCAL_ADHESION.html) | -0.54 | -1.62 | 0.008 | 0.086 | 0.439 | 5060 | tags=47%, list=15%, signal=55% |
| **TBC1D9** | | | | | | | | |
| **Follow link to MSigDB** | **GS DETAILS** | **ES** | **NES** | **NOM p-val** | **FDR q-val** | **FWER p-val** | **RANK AT MAX** | **LEADING EDGE** |
| [KEGG_HEDGEHOG_SIGNALING_PATHWAY](http://www.gsea-msigdb.org/gsea/msigdb/cards/KEGG_HEDGEHOG_SIGNALING_PATHWAY) | [Details ...](file:///C:\..\2019%25E5%2590%25B4%25E6%25A2%25A6%25E7%2591%25B6-paper\%25E7%2594%259F%25E4%25BF%25A1%25E5%2588%2586%25E6%259E%2590\%25E7%25BB%2593%25E6%259E%259C\%25E7%25BA%25BF%25E7%25B2%2592%25E4%25BD%2593\GSEA_RESULTS\TBC1D9_KEGG.Gsea.1622386604476\KEGG_HEDGEHOG_SIGNALING_PATHWAY.html) | 0.6 | 1.66 | 0 | 0.321 | 0.086 | 8568 | tags=55%, list=26%, signal=74% |
| [KEGG_T_CELL_RECEPTOR_SIGNALING_PATHWAY](http://www.gsea-msigdb.org/gsea/msigdb/cards/KEGG_T_CELL_RECEPTOR_SIGNALING_PATHWAY) | [Details ...](file:///C:\..\2019%25E5%2590%25B4%25E6%25A2%25A6%25E7%2591%25B6-paper\%25E7%2594%259F%25E4%25BF%25A1%25E5%2588%2586%25E6%259E%2590\%25E7%25BB%2593%25E6%259E%259C\%25E7%25BA%25BF%25E7%25B2%2592%25E4%25BD%2593\GSEA_RESULTS\TBC1D9_KEGG.Gsea.1622386604476\KEGG_T_CELL_RECEPTOR_SIGNALING_PATHWAY.html) | 0.62 | 1.53 | 0.011 | 0.467 | 0.35 | 8727 | tags=67%, list=26%, signal=90% |
| [KEGG_CHEMOKINE_SIGNALING_PATHWAY](http://www.gsea-msigdb.org/gsea/msigdb/cards/KEGG_CHEMOKINE_SIGNALING_PATHWAY) | [Details ...](file:///C:\..\2019%25E5%2590%25B4%25E6%25A2%25A6%25E7%2591%25B6-paper\%25E7%2594%259F%25E4%25BF%25A1%25E5%2588%2586%25E6%259E%2590\%25E7%25BB%2593%25E6%259E%259C\%25E7%25BA%25BF%25E7%25B2%2592%25E4%25BD%2593\GSEA_RESULTS\TBC1D9_KEGG.Gsea.1622386604476\KEGG_CHEMOKINE_SIGNALING_PATHWAY.html) | 0.58 | 1.52 | 0.006 | 0.392 | 0.362 | 7991 | tags=55%, list=24%, signal=72% |
| [KEGG_CYTOKINE_CYTOKINE_RECEPTOR_INTERACTION](http://www.gsea-msigdb.org/gsea/msigdb/cards/KEGG_CYTOKINE_CYTOKINE_RECEPTOR_INTERACTION) | [Details ...](file:///C:\..\2019%25E5%2590%25B4%25E6%25A2%25A6%25E7%2591%25B6-paper\%25E7%2594%259F%25E4%25BF%25A1%25E5%2588%2586%25E6%259E%2590\%25E7%25BB%2593%25E6%259E%259C\%25E7%25BA%25BF%25E7%25B2%2592%25E4%25BD%2593\GSEA_RESULTS\TBC1D9_KEGG.Gsea.1622386604476\KEGG_CYTOKINE_CYTOKINE_RECEPTOR_INTERACTION.html) | 0.54 | 1.52 | 0.021 | 0.338 | 0.369 | 9420 | tags=55%, list=28%, signal=76% |
| [KEGG_NATURAL_KILLER_CELL_MEDIATED_CYTOTOXICITY](http://www.gsea-msigdb.org/gsea/msigdb/cards/KEGG_NATURAL_KILLER_CELL_MEDIATED_CYTOTOXICITY) | [Details ...](file:///C:\..\2019%25E5%2590%25B4%25E6%25A2%25A6%25E7%2591%25B6-paper\%25E7%2594%259F%25E4%25BF%25A1%25E5%2588%2586%25E6%259E%2590\%25E7%25BB%2593%25E6%259E%259C\%25E7%25BA%25BF%25E7%25B2%2592%25E4%25BD%2593\GSEA_RESULTS\TBC1D9_KEGG.Gsea.1622386604476\KEGG_NATURAL_KILLER_CELL_MEDIATED_CYTOTOXICITY.html) | 0.57 | 1.52 | 0.036 | 0.302 | 0.375 | 8508 | tags=54%, list=26%, signal=72% |
| [KEGG_VEGF_SIGNALING_PATHWAY](http://www.gsea-msigdb.org/gsea/msigdb/cards/KEGG_VEGF_SIGNALING_PATHWAY) | [Details ...](file:///C:\..\2019%25E5%2590%25B4%25E6%25A2%25A6%25E7%2591%25B6-paper\%25E7%2594%259F%25E4%25BF%25A1%25E5%2588%2586%25E6%259E%2590\%25E7%25BB%2593%25E6%259E%259C\%25E7%25BA%25BF%25E7%25B2%2592%25E4%25BD%2593\GSEA_RESULTS\TBC1D9_KEGG.Gsea.1622386604476\KEGG_VEGF_SIGNALING_PATHWAY.html) | 0.56 | 1.47 | 0.008 | 0.362 | 0.485 | 10137 | tags=63%, list=31%, signal=91% |
| [KEGG_NOTCH_SIGNALING_PATHWAY](http://www.gsea-msigdb.org/gsea/msigdb/cards/KEGG_NOTCH_SIGNALING_PATHWAY) | [Details ...](file:///C:\..\2019%25E5%2590%25B4%25E6%25A2%25A6%25E7%2591%25B6-paper\%25E7%2594%259F%25E4%25BF%25A1%25E5%2588%2586%25E6%259E%2590\%25E7%25BB%2593%25E6%259E%259C\%25E7%25BA%25BF%25E7%25B2%2592%25E4%25BD%2593\GSEA_RESULTS\TBC1D9_KEGG.Gsea.1622386604476\KEGG_NOTCH_SIGNALING_PATHWAY.html) | 0.61 | 1.47 | 0.027 | 0.338 | 0.489 | 8250 | tags=57%, list=25%, signal=76% |
| [KEGG_B_CELL_RECEPTOR_SIGNALING_PATHWAY](http://www.gsea-msigdb.org/gsea/msigdb/cards/KEGG_B_CELL_RECEPTOR_SIGNALING_PATHWAY) | [Details ...](file:///C:\..\2019%25E5%2590%25B4%25E6%25A2%25A6%25E7%2591%25B6-paper\%25E7%2594%259F%25E4%25BF%25A1%25E5%2588%2586%25E6%259E%2590\%25E7%25BB%2593%25E6%259E%259C\%25E7%25BA%25BF%25E7%25B2%2592%25E4%25BD%2593\GSEA_RESULTS\TBC1D9_KEGG.Gsea.1622386604476\KEGG_B_CELL_RECEPTOR_SIGNALING_PATHWAY.html) | 0.63 | 1.45 | 0.011 | 0.312 | 0.523 | 7693 | tags=65%, list=23%, signal=85% |
| [KEGG_SMALL_CELL_LUNG_CANCER](http://www.gsea-msigdb.org/gsea/msigdb/cards/KEGG_SMALL_CELL_LUNG_CANCER) | [Details ...](file:///C:\..\2019%25E5%2590%25B4%25E6%25A2%25A6%25E7%2591%25B6-paper\%25E7%2594%259F%25E4%25BF%25A1%25E5%2588%2586%25E6%259E%2590\%25E7%25BB%2593%25E6%259E%259C\%25E7%25BA%25BF%25E7%25B2%2592%25E4%25BD%2593\GSEA_RESULTS\TBC1D9_KEGG.Gsea.1622386604476\KEGG_SMALL_CELL_LUNG_CANCER.html) | 0.61 | 1.44 | 0.015 | 0.315 | 0.534 | 6246 | tags=52%, list=19%, signal=64% |
